# Review of Applications of β-Cyclodextrin as a Chiral Selector for Effective Enantioseparation

**DOI:** 10.3390/ijms251810126

**Published:** 2024-09-20

**Authors:** Ewa Napiórkowska, Łukasz Szeleszczuk

**Affiliations:** 1 Department of Organic and Physical Chemistry, Faculty of Pharmacy, Medical University of Warsaw, Banacha 1 Str., 02-093 Warsaw, Poland; ewa.napiorkowska@wum.edu.pl; 2 Doctoral School, Medical University of Warsaw, Żwirki i Wigury 81 Str., 02-093 Warsaw, Poland

**Keywords:** β-cyclodextrin, binding constant, enantioselectivity, enantiomer, chiral selector

## Abstract

The significance and necessity of separating enantiomers in food, pharmaceuticals, pesticides, and other samples remains constant and unrelenting. The successful chiral separation usually includes the application of a chiral auxiliary compound, known also as a chiral selector (CS), that forms complexes with enantiomers of different physicochemical properties, enabling efficient separation. While both native and substituted cyclodextrins (CDs) are commonly used as CSs, β-CD is undoubtedly the most popular one among them. This review includes recent advancements in the application of β-CD as a CS. While the theoretical background behind the enantioseparation is also part of this work, the main emphasis is put on the factors that affect the efficacy of this process such as temperature, pH, solvent, and the choice of other additives. Also, the different analytical methods: Nuclear Magnetic Resonance (NMR) spectroscopy, Capillary Electrophoresis (CE), fluorescence spectroscopy (FS), High-Performance Liquid Chromatography (HPLC), Isothermal Titration Calorimetry (ITC), and UV–vis spectroscopy, used for enantioseparation with the aid of β-CD as CS, are thoroughly compared. Also, since some of the chiral compounds have been studied in the context of their enantioseparation more than once, those works are compared and critically analyzed. In conclusion, while β-CD can be in most cases used as CS, the choice of the experimental conditions and method of analysis is crucial to achieve the success.

## 1. Introduction

In 1848, Louis Pasteur, who is known for his contributions to chirality-concerned studies, first proposed the concept of stereoisomerism after noticing the distinct visual properties of mechanically separated tartrate enantiomers [1]. The characteristic features of a pair of enantiomers are identical chemical composition and physicochemical properties of those two molecules. Nevertheless, in a chiral environment, they exhibit different interactions with other molecules, such as chiral selectors, owing to the varying spatial arrangement of the substituent groups around one or more asymmetric centers.

The distinct impacts of enantiomers and their varying behaviors in biological processes can be observed at a macroscopic level. For instance, L-proline has a sweet component and D-proline is bitter [2], the (S)-enantiomer of an active pharmaceutical ingredient-naproxen has shown 28 times higher pharmacological activity in clinical studies [3], whereas the (R)-enantiomer is hepatotoxic. Therefore, according to the European Pharmacopeia (EurPh) regulations, the single (S)-isomer should be used with a chiral impurity of up to 2.5% [4].

The significance and necessity of separating enantiomers present in samples of food, pharmaceuticals, pesticides, and insecticides remains constant and unrelenting. The investigation of enantiomers found in food products has garnered significant interest, particularly about their genuineness, legality, and safety. For example, the natural products of fruit juices should exclusively contain L-amino acids. The presence of D-amino acids can indicate adulteration or other issues that may have happened during inadequate storage [5]. Pharmaceutical applications involve the use of a wide range of chiral drugs in humans. It is possible that only one of the two enantiomers exhibits the desired effects, known as the eutomer, while the other enantiomer, called the distomer, is less effective or may even cause undesirable or harmful side effects, like the thalidomide example whose (R)-enantiomer is responsible for the sedative effect whereas (S)-isomer can cause birth defects [6]. The occurrence of this phenomenon can be attributed to the variations in selective interactions with the chiral biological matrices found in living beings during biochemical processes. Additionally, enantiomeric substances can display characteristic pharmacokinetics and pharmacodynamics. Ensuring the enantiomeric purity of pharmaceuticals is a critical concern in clinical and analytical research, as well as for regulatory considerations [7]. The pursuit of stereoselective synthesis, which aims to produce a single enantiomer with high yield and improved enantiomeric/diastereomeric excess (ee, de), has garnered significant interest. However, those methods have some limitations such as harsh reaction conditions, generation of side products, and high toxicity of reagents. Hence, the process of separating enantiomers, known as enantiomeric separation, has garnered attention in various disciplines such as pharmaceuticals, biomedicine, agrochemicals, and food sciences, among others [8].

Cyclodextrins (CDs) are a group of cyclic oligosaccharides that are made up of glucose subunits connected by α-1,4 glycosidic bonds. For example, β-cyclodextrin (β-CD), which is the object of this review, is composed of seven glucose subunits. Since the 1970s, there has been a significant surge in the interest in the utilization of CDs in industrial applications [9]. The insight at the molecular level can provide the explanation for the favorable features of CDs in the enantiomeric separation. CD molecules have a ring-like structure similar to a doughnut, allowing them to trap other substances. When a non-polar molecule, such as a poorly soluble API, enters the molecular cavity of cyclodextrin, it forms a host–guest complex. This complex is polar on the outside, and, as a result, it is more soluble than the free guest molecule in aqueous medium. Since the binding constants of the formed inclusion complexes depend on the structure of the guest molecule, it is likely that the complexes of enantiomers would be characterized by different stabilities.

This review includes recent advancements in β-CD application as a chiral selector. Although more general reviews on the application of various CDs and CD-based materials in the field of enantioseparation (ES) have been published [10,11,12,13], the current one focuses solely on native β-CD, being the most widely applicable one. The structure of this review presents as follows. First, the theoretical background of enantioseparation is summarized, introducing the most important definitions and concepts as well as recent insights into the mechanism of chiral recognition. Then, the methods commonly used to determine the binding constants are presented and compared. In the next chapter, chosen factors influencing the effectiveness of β-CD as a chiral selector are discussed, based on the recently published studies. Since the β-CD-assisted ES of some of the chiral compounds, due to their high significance, have been studied in multiple works, the next chapter compares the results of those studies.

## 2. Cyclodextrins as Chiral Selectors—Why β-CD Is the Most Popular One

**Figure 1:**
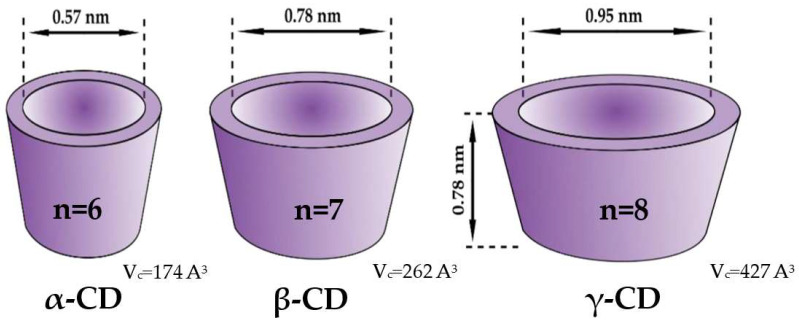
Schematic structure of native α, β, and γ-CD; n—number of glucose units, V_c_—cavity volume. Reprinted with permission from [19], licensed under CC BY 4.0.

While both native and substituted CDs are commonly used as chiral selectors, β-CD is undoubtedly the most popular one among them. Furthermore, ES is not the only field that was dominated by this particular CD. For example, a comprehensive comparison and review of pharmacological formulations containing CDs as excipients revealed that β-CD is the most often used one [14], with 54.8% of the market, while the second one, HP-β-CD, scored only 16.1%. The rationale behind this can be attributed to the simplicity of its manufacturing process and the resulting affordability, with an annual production exceeding 10,000 tons and an average wholesale price of around 5 USD per kilogram [15]. Whilst those practical features have undoubtedly contributed to the high popularity of β-CD among the other ones, this widespread use would be impossible without the desirable molecular properties of this host molecule. For example, the CDs differ in their sizes, including the inner diameter (ID) and cavity volume (V_c_) (Figure 1). In the case of native CDs, α-, β-, and γ-CD possess the ID of 5.7 Å, 7.8 Å, and 9–10 Å, respectively. The cavity diameter of β-CD was found to be ideal for accommodating molecules of hormones, vitamins, and many other chemicals often employed for various applications. Additionally, the half-life (t_1/2_) for the ring-opening of β-CD in pure aqueous solutions was found to be around 15 hours at a temperature of 70 °C and a pH of 1.1, according to a study by Hirayama et al. [16]. According to Schönberger et al., β-CD is about 1.5 times more stable than γ-CD [17]. Therefore, β-CD is mostly utilized as a complexing agent. This domination is also reflected in the number of crystal structures of the complexes of native CDs deposited in CCDC, being equal to 131, 196, and 430 for γ-, α-, and β-CD, respectively [18].

### Crystal Structures of The Complexes with Native Β-CD

**Figure 2:**
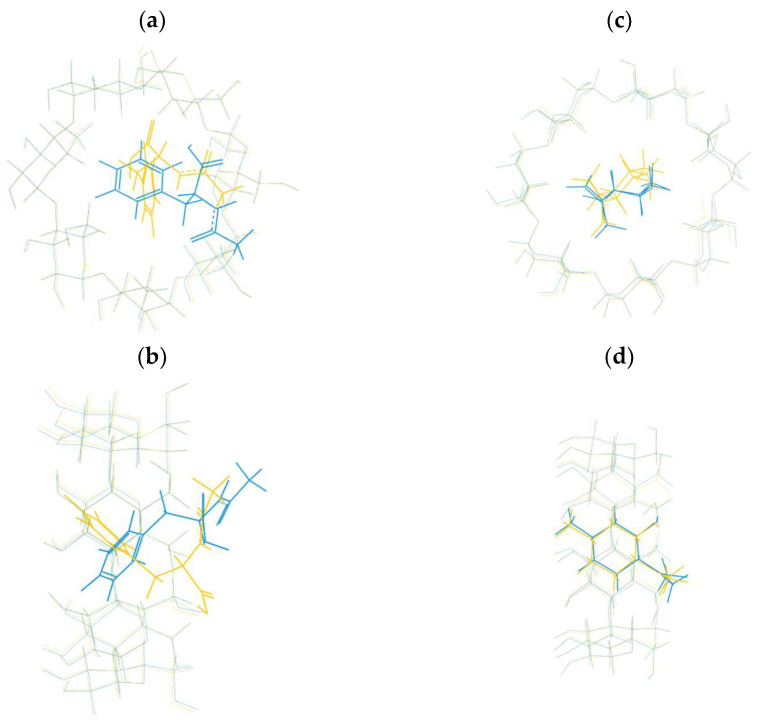
A superposition of inclusion complexes with β-CD and (**a**,**b**) N-acetyl-L-phenylalanine (yellow) and N-acetyl-D-phenylalanine (blue); (**c**,**d**) (+)-isopulegol (yellow) and (-)-isopulegol (blue).

Although β-CD can form stable complexes with various guests, a limited number of complexes have been registered in CCDC with both enantiomers, allowing the comparison of their crystal structures. In Figure 2, examples of the formation of the structurally comparable and distinct complexes between β-CD and the enantiomers of the same compound have been presented. The inclusion complexes of N-acetyl-D-phenylalanine (REFCODE: AGAZUD [20]), N-acetyl-L-phenylalanine (AGAZIR [21]), and (+)-isopulegol (PAKREB [22]), (-)-isopulegol (PAKRAX [23]) with β-CD as a host have been superimposed. Even though the conformations of CD in both examples overlap completely, in the case of N-acetyl-phenylalanine enantiomers, the conformation of guests significantly differs between D- and L-isomer (Figure 2a,b). A detailed comparison of the crystal structures can be found in [24]. Likewise, significant differences in the orientation of the guest molecules within the cavity have been observed in the case of fenoprofen stereoisomers [25]. In contrast, the conformations of (-)- and (+)-isopulegol inside the β-CD cavity seem isostructural (Figure 2c,d), and the observation has been supported by HPLC analysis [26]. Similarly to isopulegol enantiomers, no significant discrepancies have been registered between the crystal structures of the inclusion complexes of the borneol enantiomers [27].

## 3. Aim and Design of the Study

Two independent examiners (E.N. and Ł.S.) were selected to choose the articles for the purpose of this review. The examiners performed a comprehensive literature search regarding the enantioselectivity of β-CD in the Scopus and PubChem databases. The following keywords and their synonyms were included among the search terms: “binding constant”, “binding affinity”, “enantioselectivity”, “chiral selector” and “β-cyclodextrin” This review aims to gather and analyze the works in which chiral recognition using native β-CDs have been investigated in terms of affinity constant calculations.

**Figure 3:**
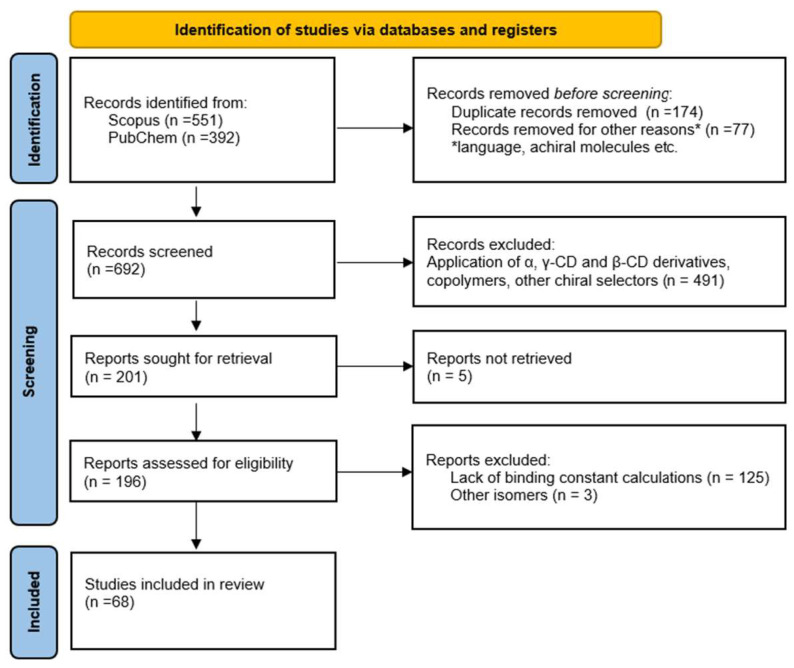
Flow diagram according to PRISMA statement.

Hence, the inclusion criteria for this review were the application of native β-CD as a chiral selector and the calculated binding constant for each enantiomer. The exclusion criteria for this study were the use of solely other types of cyclodextrins, i.e., α-, γ-CD, or β-CD derivatives, application of β-CD to separate different stereoisomers from enantiomers, e.g., cis-, trans-, M-, or P- isomers or the lack of information on binding constants, or the values of Gibbs Free energy (ΔG). After finishing the inclusion and exclusion process, any disagreements were handled by the consensus between the reviewers. A thorough review has been conducted of the included publications to identify any further relevant research that may be included in the review. The flowchart based on the Preferred Reporting Items for Systematic Reviews (PRISMA) statement [28] is presented in Figure 3.

**Figure 4:**
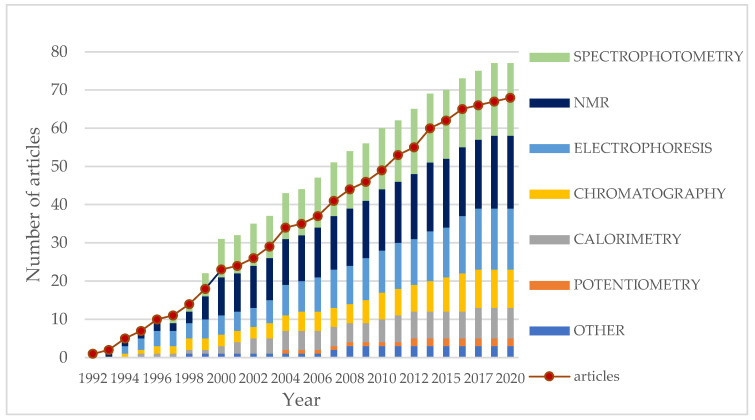
Number of published studies on the application of β-CD as a chiral selector in which the enantioselectivity has been quantitively assessed. The dot plot represents the number of articles published up to the particular year. Each column displays the cumulative number of application β-CD as chiral selectors for the specified year and all previous years divided based on the category of analytical methods used to determine binding constants.

Figure 4 presents the categorization of the included articles based on the applied analytical methods to calculate binding constants (K) for enantiomers. Since in several studies more than one analytical method has been applied for binding constant calculations, the dot plot represents the cumulative number of works published up to a particular year. According to the graph, spectrophotometric and NMR analyses have been the predominant methods used to determine binding constants since 2000 (Table 1). However, electrophoresis analysis has been also widely used for this purpose. The investigated pairs of enantiomers from selected articles with the binding constant values and the conditions of analysis have been summarized and are presented in Table 2. The differences in affinity constants between enantiomers and the ratio of K values (α) have been calculated based on K values (ΔK) provided for each enantiomer, unless they were already included in the original works.

## 4. Theoretical Overview

For 1:1 stoichiometry, the equilibrium equation between the bound and unbound analyte to the chiral selector, in this case, to β-CD, can be described as follows:

(1) G+βCD⇄G−βCD

where G is the guest, and “G − βCD” is a complex formed between the analyte and β-CD. The binding constant (K) can be expressed as a ratio between equilibrium molar concentrations of complex [G − βCD], free guest [G], and [βCD]:

(2) $$K=\frac{\left[G-\beta CD\right]}{\left[G\right]\left[\beta CD\right]}$$

And it is the inverse of the dissociation constant (K_d_). Due to the law of conservation of mass assuming any adsorption of guest and host molecules, the formula can be written as:

(3) $$K=\frac{\left[G-\beta CD\right]}{\left({\left[G\right]}_{0}-\left[G-\beta CD\right]){(\left[\beta CD\right]}_{0}-\left[G-\beta CD\right]\right)}$$

which after transformations to the concentration of the formed complex between guest and β-CD ([G − βCD]), required for substitution of [G − βCD] in Equations (5), (6), and (12), the real solution of Equation (3) can be as follows:

(4) $$\left[G-\beta CD\right]=\frac{\left.\left.{\left([G\right]}_{0}+{\left[\beta CD\right]}_{0}+\frac{1}{K})-\sqrt{{\left({\left[G\right]}_{0}+{\left[\beta CD\right]}_{0}+\frac{1}{K}\right)}^{2}-4{\left[G\right]}_{0}{\left[\beta CD\right]}_{0}}\right.\right.}{2}$$

Based on this Equation (3), if the $\left[G\;-\;\beta CD\right]$ and [G]_0_, [βCD]_0_ are known, the binding constant can be exactly calculated. Unfortunately, the difficulty in direct measurement of [G − βCD] in supramolecular chemistry leads to indirect methods to monitor the concentration of formed complexes, i.e., titration [97], in which the concentration of one of the components, e.g., host [βCD]_0_, is kept constant with increasing the concentration of guests and monitoring the change in physical properties.

The change in physical property (Y) can be typically described as an aggregate of the physical properties of the guest (Y_G_), host (Y_βCD_), and the complex (Y_G-βCD)._ The physical property of interest can vary and be related to the applied methods, i.e., electrophoretic mobility in EC, chemical shift (δ) in NMR, absorbance in UV–vis spectroscopy, heat absorbed/released (Q) in calorimetry, etc. If the observed change is dependent on the concentration, such as in the case of UV–vis titration, the function can be formulated as:

(5) $$Y=Y_{G}{\left[G\right]}_{0}+Y_{\beta CD}{\left[\beta CD\right]}_{0}+Y_{G-\beta CD}[G-\beta CD]$$

If the titration analysis starts without the guest, the change in the physical property after increasing the concentration of β−CD can be defined as:

(6) $$∆Y=Y_{G}{\left[G\right]}_{0}+Y_{∆G-\beta CD}[G-\beta CD]$$

where

(7) $$Y_{\Delta G-\beta CD}={Y_{G-\beta CD}-Y}_{\beta CD}$$

And after substitution of $\left[G-\beta CD\right]$ from the Equation (4):

(8) $$∆Y=Y_{G}{\left[G\right]}_{0}+Y_{∆G-\beta CD}\left(\frac{\left.\left.{\left([G\right]}_{0}+{\left[\beta CD\right]}_{0}+\frac{1}{K})-\sqrt{{\left({\left[G\right]}_{0}+{\left[\beta CD\right]}_{0}+\frac{1}{K}\right)}^{2}-4{\left[G\right]}_{0}{\left[\beta CD\right]}_{0}}\right.\right.}{2}\right)$$

In Equation (8), the [G]_0_ and [βCD]_0_ are the initial concentrations of guest and β-CD, respectively, and they are both known; however, the K and $Y_{∆G-\beta CD}$ variables remain unknown. The unknown variables, i.e., K and $Y_{∆G-\beta CD}$, and $Y_{G}$ can be obtained by using nonlinear regression analysis by fitting the results data to Equation (8) using suitable software. It is worth mentioning that $Y_{G}$ can be obtained by performing separate analyses for guests solely and measuring the physical property of interest.

For example, in the case of UV–vis titration, the physical property of interest is absorbance (A) according to the Beer–Lambert law (A=εcl, where A—absorbance, ε—molar absorption coefficient, c—molar concentration, l—optical path length). The change in absorbance (ΔA) can be described as:

(9) $$∆A=\varepsilon_{G}{\left[G\right]}_{0}+\varepsilon_{∆G-\beta CD}\left(\frac{\left.\left.{\left([G\right]}_{0}+{\left[\beta CD\right]}_{0}+\frac{1}{K})-\sqrt{{\left({\left[G\right]}_{0}+{\left[\beta CD\right]}_{0}+\frac{1}{K}\right)}^{2}-4{\left[G\right]}_{0}{\left[\beta CD\right]}_{0}}\right.\right.}{2}\right)$$

where the molar absorptivity of the guest ($\varepsilon_{G})$ can be obtained by separate measurements of the absorbance for a guest solution without the presence of a host or by including this variable as an unknown parameter in nonlinear fitting analysis.

Since some physical properties like the chemical shift (δ) in NMR analysis depend on the mole fraction (f), the aggregate of physical properties can be formulated as:

(10) $$Y=Y_{\beta CD}f_{\beta CD}{+Y}_{G}f_{G}+Y_{G-\beta CD}f_{G-\beta CD}$$

And the mole fraction of complex:

(11) $$f_{G-\beta CD}=\frac{\left[G-\beta CD\right]}{{\left[\beta CD\right]}_{0}}=\frac{K\left[G\right]}{1+K[G]}$$

The change in physical property depending on mole fraction can be described:

(12) $$∆Y=Y_{∆G-\beta CD}\left(\frac{\left[G-\beta CD\right]}{{\left[\beta CD\right]}_{0}}\right)$$

(13) $$∆Y=\frac{Y_{∆G-\beta CD}}{{\left[\beta CD\right]}_{0}}\left(\frac{\left.\left.{\left([G\right]}_{0}+{\left[\beta CD\right]}_{0}+\frac{1}{K})-\sqrt{{\left({\left[G\right]}_{0}+{\left[\beta CD\right]}_{0}+\frac{1}{K}\right)}^{2}-4{\left[G\right]}_{0}{\left[\beta CD\right]}_{0}}\right.\right.}{2}\right)$$

The physical property of interest in NMR analysis is chemical shift, and the change to the addition of guests can be presented as:

(14) $$\Delta \delta =\delta {-\delta }_{0}$$

(15) $$\delta_{\Delta G-\beta CD}={\delta_{G-\beta CD}-\delta }_{\beta CD}$$

(16) $$∆\delta =\frac{\delta_{∆G-\beta CD}}{{\left[\beta CD\right]}_{0}}\left(\frac{\left.\left.{\left([G\right]}_{0}+{\left[\beta CD\right]}_{0}+\frac{1}{K})-\sqrt{{\left({\left[G\right]}_{0}+{\left[\beta CD\right]}_{0}+\frac{1}{K}\right)}^{2}-4{\left[G\right]}_{0}{\left[\beta CD\right]}_{0}}\right.\right.}{2}\right)$$

$\delta_{0}=\delta_{\beta CD}$ as the initial analysis is performed without the addition of a guest.

The NMR and UV–vis spectroscopies have been known as the most common methods of determining the binding constant from titration experiments. For more information including calculations of association constant based on the results from different analytical techniques and consideration of stoichiometry of complexes 1:2 and 2:1, one may refer to Pall Thordarson’s paper [97].

## 5. Determination of Binding Constant

Determination of binding constant is, in the case of CD inclusion complexes, not a straightforward task, and many aspects should be taken into consideration. Therefore, Pall Thordarson in his review [98] has provided a tutorial including dos and don’ts to obtain good-quality credible data. The main findings from this extensive work have been mentioned here.

First, it is crucial to gather as much preliminary information about the tested compounds and suitable methods of analysis as possible, to design the conditions and the process of analysis thoroughly. Advancements in available software enable to perform nonlinear regression analysis easily; therefore, this approach should be used to ensure the reliability of the results. In some works, the linear approach such as the Benesi–Hildebrand method has been utilized based on the findings that, e.g., 100 times excess of chiral selector allows to obtain accurate results, with an error of 1% [99]. However, nowadays nonlinear methods are not difficult to calculate using available modern software; therefore, the linear approaches should not be used anymore [98]. Presumably, in some cases that applied linear regression, it would be beneficial to recalculate the results to obtain more precise and accurate results. In the determination of the K value, the repetition of analysis, hence the estimated uncertainty, can dramatically change the utility of the obtained data, leading to different conclusions from the analysis. 

For further guidelines on performing titration experiments and determining association constants from them, one should refer to this tutorial review [98]. In the next work [97], Thordarson has gathered the fundamentals of determining association constants in supramolecular chemistry, and part of the considerations have been presented in this review in Section 4, called “Theoretical overview”. His work has explored theoretical principles based on several analytical methods and the stoichiometry of the complexes generated, offering valuable guidance for conducting accurate analysis.

### 5.1. Binding Selectivity (α)

To quantitively assess the enantioselectivity, the ratio of binding constant has been determined as:

(17) $$\alpha =\frac{K_{H}}{K_{L}}$$

where K_H_ and K_L_ are the binding constants for the preferred enantiomer (with a higher K value) and antipode (with a lower K value), respectively. If α = 1, both enantiomers bind equally to β-CD. If α > 1, there is an indication for enantioselectivity, since both enantiomers tend to bind differently to β-CD.

There are no unified names for α factor, and among reviewed studies, the name of the α factor varied, e.g., enantioseparation factor [92,95], binding selectivity [29,48], chiral selectivity [100], enantioselectivity [83], and ^1^H NMR discrimination ratio [30]. Additionally, it is important to note that the α factor can be also defined as the ratio of retention factors, rather than the ratio of binding constants [49,75]. Therefore, it is necessary to verify the specific definition of α value being used in a particular work.

Unfortunately, there has been no established standard threshold of α values above which effective chiral selection can be defined since chiral recognition can vary depending on, e.g., sensitivity, physical property that is measured, and different mechanisms of the applied analytical methods. For instance, in [83], the enantioselectivity was obtained when α > 1.11; however, in [49], the chiral recognition was observed for brompheniramine where α = 1.05, whereas in [30], to predict enantioselectivity, the chiral discrimination α values should be above 1.30, and for α below 1.20, enantioselectivity would not be achieved.

In the chromatographic methods the baseline separation can be achieved when the resolution factor (R_s_) is above 1.5 [101]. However, the R_s_ depends not only on the retention time of the analyte, which is related to the strength of interaction with the stationary phase but also on the width of peaks that may be related to the parameters of the analysis.

(18) $$R_{s}=2\frac{t_{R2}-t_{R1}}{W_{1}+W_{2}}$$

where $t_{R1}$ and $t_{R2}$ are the retention times of the first and the second peaks, and W_1_ and W_2_ are the baseline widths of the first and the second peaks, respectively.

Therefore, the adjustment of the initial settings like flow rate, temperature, pH, ion strength, and solvent composition may enhance the ability of β-CD as a chiral selector. Nevertheless, the variations in the strength of the interaction between enantiomers and β-CD, manifesting in differences in K values, play a crucial role in the chiral recognition process.

Thermochemical analysis of differences in binding constant is governed by the equations:

(19) ΔG=−RTlnK

(20) ΔΔG=−RTlnα

where ΔG—Gibbs free energy, R—gas constant with a value of 8.314 J K^−1^ mol^−1^, T—temperature in Kelvin.

Based on the example provided in [98], if α = 2, the absolute difference in Gibbs free energy (ΔΔG) is ca. 1.7 kJ/mol, and with the increment of the K value from 50 to 100 M^−1^, there is an 18% change in terms of free energy. However, if the K value is 5 × 10^5^ M^−1^, the doubling of K tends to change in energy only by 5%. Hence, the magnitude of the binding constants can be fundamental in determining differences in complex stability and strength of interactions between enantiomers and β-CD.

### 5.2. Analytical Methods Used for Determination of Binding Constant

Among the reviewed works, various analytical methods have been employed to assess the enantioselectivity of β-CD. The most important aspects like sensitivity, complexity, time of analysis, and spectrum of molecules that can be tested using the particular method have been summarized in Table 1, with suitable examples of studies. The comparison of the techniques has been performed based on the physicochemical principles of the methods [102], the introductions of the reviewed articles, and the outcomes from the studies. Choosing an appropriate method for establishing association constant relies on several factors, such as the physicochemical properties of molecules, quantity and purity of the sample, required equipment, time and costs intended for the analyses.

The presented techniques can be divided into two groups: separation-based and non-separation-based methods [103]. The separation-based methods include chromatography (e.g. HPLC, GC), capillary electrophoresis (CE), and ultrafiltration, while the non-separation-based techniques are spectroscopy-based analysis (e.g. UV–vis, NMR, CD, and FS), potentiometry, and isothermal titration calorimetry (ITC). Compared to HPLC, CE is usually a more efficient, less time-consuming, and low-cost method since it requires lower reagent consumption and less complex equipment [49,50]. The main advantages of NMR measurement are that it can provide detailed structural information about complexes, and the analysis can be performed for the racemic mixture, resulting in time reduction in comparison to titration analysis for separated enantiomers [100]. Moreover, NMR analysis could be applied to predict the HPLC and CE results.

Performing isothermal titration calorimetry (ITC) measurement allows the determination of the thermodynamics of molecular interaction. Hence, it provides details of the enthalpy and entropy changes upon complexation and enables the direct determination of thermodynamic binding constants. It is worth noting that apparent binding constants obtained from other analytical methods under certain conditions provide information regarding the equilibrium state of the binding process but without insight into the thermodynamic properties. Therefore, calculated thermodynamic and apparent binding constants can differ [50]. Hence, in some studies, multiple approaches have been used to determine association constant and results compared to each other to obtain more accurate findings and minimize the impact of several factors on the determination of binding constraints, which will be discussed in detail in the subsequent parts of this review.

## 6. Overview of the Determination of the Binding Constants in Chiral Selective Analytical Analysis

Due to the unique properties of β-CD, described in Section 1, it has found wide application as a chiral selector in various analytical methods, and its effectiveness has been tested on many compounds, usually APIs. In numerous works, detailed information about the complexation constants for particular isomers has been provided. However, in some of the studies, the authors have not determined the affinity constants, but solely focused on the differences in the spectra, allowing the assessment of whether enantioselectivity by application of β-CD has occurred [104,105,106].

In this paragraph, we gathered and analyzed the studies in which beyond solely the detection or lack of enantioselectivity, the authors have determined the affinity of the enantiomers towards native β-CD tested on numerous compounds and at various conditions of analysis. We gathered examples of studies where affinity constants have been calculated, with a distinction made according to the method used for the determination, i.e., HPLC, CE, NMR, ITC, CD, UV, and FS measurements. In the next paragraph, the key factors that can influence the effectiveness of chiral recognition have been analyzed, with the chosen examples described in a more detailed way.

### 6.1. Liquid Chromatography (LC)

The approach with a chiral mobile phase in HPLC requires an achiral chromatographic column and an appropriate concentration of β-CD added to the working solution. This results in the formation of complexes with various complexation constants, depending on the particular isomer, which affects the retention time and allows the chiral separation. This method has been tested, e.g., in the study [49] in which chiral recognition has been observed for brompheniramine and cyclopentolate, supported by the α values of 1.05 and 1.33, respectively.

In the next study [77], the authors performed reversed-phase HPLC with the mobile phase enriched with β-CD to determine the stability constant of complexes formed between α-terpineol enantiomers. Since the obtained values of binding constants for (+)-α-terpineol (413 ± 10 M^−1^) and (-)-α-terpineol (399 ± 8 M^−1^) did not differ much, the separation by β-CD was not possible, and the authors concluded that the native β-CD cannot be used as a chiral agent to separate enantiomers of α-terpineol.

The application of β-CD as a chiral selector is not solely limited to its use in the mobile phase. It can also be employed as a chiral stationary phase (CSP) by forming covalent bonds, usually with silica particles [107,108]. Permethrin is an important API, existing in four isomers, that can be grouped into two pairs (cis and trans), with two enantiomers (R and S) in each pair. The reverse-phase HPLC analysis using a β-CD-coated column has been performed for permethrin isomers [79], in which successful enantioseparation has been obtained for both cis- and trans-pairs of enantiomers. Additionally, the authors have explained their results on the basis of thermodynamics, determining the Gibbs free energy values of complex formation, proving that cis-pair is characterized by more negative ΔG, indicating the formation of more stable complexes with β-CD than the trans-pair. This statement was further supported by the fact that the cis-pair enantiomers have shown longer retention times, due to the stronger interactions between analyte and stationary phase. It has also been declared that the separation process is enthalpy-driven. Since ΔH values differ more for R/S in the cis-pair, better chiral recognition has been observed for cis-pair compared to trans-enantiomer pair. Additionally, the temperature and solvent impact has been studied to obtain optimal conditions for enantiodiscrimination of permethrin enantiomers.

In the next work, describing the HPLC analysis [80], the chiral recognition of synthetic tetrahydronaphthalenic derivatives, potential melatoninergic compounds, has been investigated via the application of chiral stationary phase (CSP) with immobilized β-CD and β-CD added to the mobile phase. Subsequently, both approaches have been compared. It has been concluded that an enantiomer that creates a more stable complex with β-CD elutes faster in the approach of the chiral mobile phase due to the formation of the weak interactions with stationary phase C18. The addition of β-CD to the mobile phase reduces the retention time of solutes, with the increase in the resolution. Contrary, if β-CDs are incorporated into the stationary phase (CSP), the enantiomer forming stronger interaction with β-CD retains longer, causing a reversal of elution order between both attempts. The effects of temperature and composition of mobile phase on retention and stereoselectivity have also been investigated for chiral stationary phase systems. It has been observed that an increase in temperature leads to a reduction in resolution, selectivity, and retention factor. Furthermore, the increase in methanol concentration from 1 to 5% leads to a decrease in the retention factor and resolution for all 3 tested compounds. Therefore, condition optimization is a crucial step in determining enantioselectivity. In the case of the chiral mobile phase approach, the concentration of β-CD has a major impact on resolution and retention time, leading to almost 1.5 times lower values of retention time with the increase in concentration from 15 to 25 mM.

### 6.2. Capillary Electrophoresis (CE)

The separation of the enantiomers in capillary electrophoresis depends strongly on differences in binding affinities of analyte enantiomers toward chiral selectors, resulting in different association constants of diastereomeric complexes [63]. However, there are reported studies claiming that the differences in electrophoretic mobilities play a crucial role [50,55]. However, in most cases, the differences have been observed in both binding constant values and mobilities. In the case of sibutramine (SIB) [48], it has been noted that the retention time of a particular isomer depended on the concentration of β-CD. Below 10 mM, (S)-SIB enantiomer migrated first; however, if β-CD concentration was greater than 10 mM, (R)-SIB retention time was shorter. The migration order and the degree of enantioselectivity were related to the relative magnitude of binding constant and complex mobility with the increase in β-CD concentration. Therefore, the reverse of the migration order has been observed. At low concentrations of the chiral selector, most of the enantiomers in solution are unbound, hence the migration order depends primarily on the relative stability of diastereomeric complexes. At higher β-CD concentrations, most of the enantiomers molecules are bonded in the cavity of CDs forming complexes, thus the differences in the mobility of complexes play a more important role in the migration order. Additionally, the β-CD concentration not only affects the mobility of complexes but also has an impact on the viscosity of the buffer. Interestingly, at 10 mM, a single peak has been observed in the electropherogram, suggesting that the binding and migration selectivity counterbalance each other, and no enantioseparation has been detected.

In another CE experiment [51], the authors investigated the enantioselection of Tröger’s base (TB) enantiomers. It is worth mentioning that the chirality of TB is not related to asymmetric carbon in its structure but to two bridgehead stereogenic nitrogen atoms. The capillary electrophoresis analyses were conducted at pH 2.5, at which TB is positively charged, with the application of α, β, γ-CD, and their carboxymethyl derivatives. Based on the results, among native cyclodextrins, β-CD had the highest binding constant, indicating the formation of a stable complex with TB. Additionally, for carboxymethyl derivatives, it was noticed that the change even in one carboxymethyl group position on one single glucose unit can significantly influence the value of a binding constant. Hence, it was concluded that the position of the carboxymethyl group in monocarboxymethyl-α, β, γ-CD and the size of the cavity played a significant role in the enantioseparation of a racemic mixture of TB.

In the next work [52], the apparent binding constants of complexes of acyclic nucleoside phosphonates (ANPs), a group of antiviral drugs, with β-CD were determined using affinity capillary electrophoresis (ACE) analysis. Based on the results, the differences in strength of complexation of R- and S-isomers allowed to distinguish enantiomers and, except for ANP 4 compound, accomplished baseline separation, regardless of relatively small values of binding constants, placed in the range 13.3–46.4 M^−1^. It was also observed that the mobilities of ANPs-β-CD complexes differed more significantly compared to electrophoretic mobilities of free enantiomers, for which values were similar between tested ANPs. Hence, the differences in strength of non-covalent interactions, including hydrogen bonds, van der Waals forces, and dipole–dipole interactions, in diastereomeric complexes between enantiomers affected the changes in mobility, leading to enantioseparation.

To conclude, the two main principles explain the separation of enantiomers using chiral selectors in capillary electrophoresis (CE) [50]. The first assumes that the strength of interaction, values of binding constant and ΔK between two enantiomers are responsible for enantioseparation, while the second elucidates enantioseparation by different mobilities of the temporarily formed complexes between analytes. In most cases, such as for the phenethylamine enantiomers [50], although the diversified mobility of complexes has been indicated to have a significant role in chiral discrimination, the values of ΔK additionally influence the efficiency of the enantioseparation.

### 6.3. Nuclear Magnetic Resonance (NMR)

The application of NMR analysis allows the detection of host–guest interaction without the need to separate enantiomers. Therefore, this approach has a wide application in chiral detection analysis.

The chiral recognition of ketoconazole (KC) enantiomers in the presence of (+)-L-tartaric acid has been investigated using NMR analysis supported by theoretical calculations [43]. The obtained values of binding constants were based on the changes in the chemical shift of the selected proton. Its signal splits upon β-CD addition, indicating the differences in complex stability between enantiomers, allowing chiral recognition. It has been remarked that the addition of a third component, i.e., (+)-L-tartaric acid, not only increases the solubility of KC, β-CD, and their complexes but also plays a crucial role in the chiral recognition process by establishing electrostatic interactions of the 2- and/or 3-hydroxyl group of β-CD with the imidazole ring of KC. The molecular modeling approach helped to define the most likely structures of complexes, and computational results were in good agreement with the experimental ones. It has been suggested that the model in which the dichlorophenyl moiety is included in the cavity from its wider rim represents the most stable structure of complexes, confirmed with the 2D NOE cross-peak intensities. Lastly, it has been noticed that the phenoxy fragment serves as a third contact site, engaged in van der Waals and electrostatic interactions with the wider rim of β-CD, with the possibility of hydrogen bond formation. All three interaction sites were responsible for the differences in the diastereomeric complexes for (+)- and (-)-KC, leading to chiral recognition by the application of β-CD as a chiral selector in the presence of a third component.

The authors of the next work performed NMR analysis in order to characterize the complexes of β-CD with promethazine hydrochloride enantiomers (PTZ) [33]. The identified changes in chemical shifts and the presence of new signal in the ^1^H NMR spectra were explained by the consequences of the inclusion of the chiral carbon inside the cavity, resulting in the differences in binding constant between R- and S-enantiomers. Consequently, the observed chiral recognition was attributed to the presence of two distinct binding sites, in which β-CD influences the microenvironment of the chiral carbon, and to the rigid structures of β-CD limiting the changes in macrocyclic conformation upon complexation. The findings have followed the results obtained from FS measurements. In addition, the 2D rotating-frame Overhauser enhancement spectroscopy (ROESY) analysis, supported by molecular modeling, has allowed to propose the most likely structure of the PTZ-β-CD complex.

**Figure 5:**
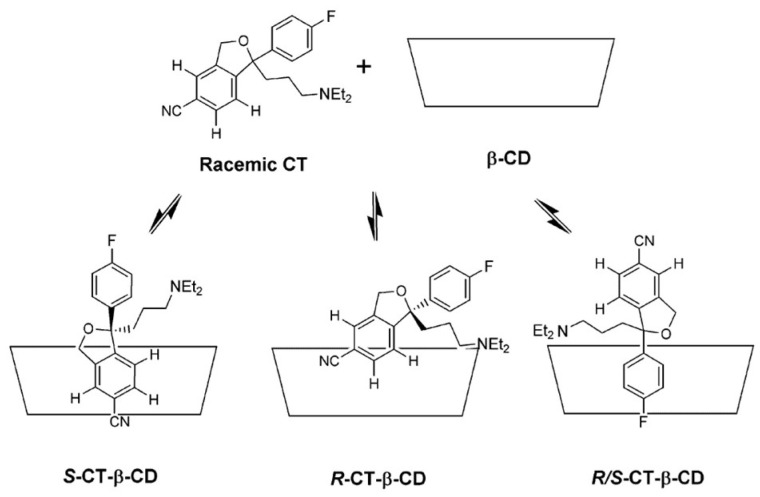
Proposed structures for the 1:1 complexes formed between (R,S)-citalopram and β-CD. Reprinted with permission from [34].

NMR spectroscopy investigation demonstrated the formation of four, structurally different, 1:1 citalopram-β-CD inclusion complexes in aqueous solution [34]. The presence of cross-peaks between the protons of both aromatic rings of citalopram (CT), with the β-CD cavity protons at the ROESY spectrum, has revealed that both aromatic rings can be incorporated inside the β-CD cavity. However, since the mode of penetration 4-fluorobenzene ring of CT has appeared to be alike between both enantiomers, the chiral discrimination can be attributed to the differences in the mode of penetration of a CN-containing ring inside the β-CD cavity (Figure 5). It has been shown that the inclusion of the CN-containing ring is only feasible for R-CT due to the hindrance caused by the dimethylaminopropyl group for S-CT. The binding constant for R-CT-β-CD and R/S-CT-β-CD complexes have been obtained using Scott’s method, yielding the values of 418 and 436 M^−1^, respectively.

In the next study [35], the inclusion complexes with a potential aromatase inhibitor have been studied by NMR analysis. The results were compared to the previous investigation of chiral recognition of chiral N-imidazole derivative (IB) using CE method [56]. Both approaches have led to the similar observation that R-IB shows a stronger affinity to β-CD than S-IB and that β-CD can be successfully utilized as a chiral selector in the case of IB [35]. Since the analysis conditions were similar between studies, i.e., 298 K at pH 2.5, the values of obtained binding constants could be compared. Although the calculated absolute K values can differ depending on the analyzed ^1^H signals, a similar pattern of changes in chemical shifts and signal splitting upon complexation has been observed regardless of the investigated signals. Upon comparing K values, it has been found that the binding constant from CE analysis has been higher than the one determined using the NMR approach. For instance, the highest values observed for R and S-IB were 242 and 157 M^−1^ for the NMR while 322 and 281 M^−1^ for CE studies, respectively. This discrepancy can be partially related to the presence of methanol, essential for dissolving the analyte in NMR solutions, which leads to competition between the analyte and solvent in the β-CD cavity. Moreover, the discrepancies may result from different measurement principles in NMR and CE studies. Nonetheless, both methods have allowed the successful chiral selection of IB enantiomers associated with the differences in α ratio of 1.54 and 1.15, for NMR and CE studies, respectively.

The ROESY analysis can support the identification of reliable structures of diastereomeric complexes. Based on one-dimensional ROESY analysis, it has been stated that in the case of native β-CD, dimethindene molecules (DIMs) penetrated deeper into the host cavity, creating more stable complexes than complexes with TM-β-CD [37]. Thus, higher affinity has been observed for R- enantiomer with application of native β-CD. Unexpectedly, those variations in structure and strength of interactions have resulted in a reversed order of R- and S enantiomers when native and derivate of β-CD were employed, supported by CE analysis. It has also been remarked from ESI-MS analysis that in solution, the 1:1 DIM-β-CD complex predominantly exists; however, they may also form complexes with a stoichiometry 1:2.

NMR analysis can be used to predict the effectiveness of β-CD as a chiral selector in reverse-phase HPLC (RP-HPLC) analysis; however, several factors need to be taken into consideration [30]. Performing chiral recognition analysis can offer advantages such as not requiring the separation of enantiomers or selection of optimal conditions, e.g., the composition of the mobile phase and the analysis can be time-consuming. Additionally, some compounds can be sensitive to UV light, which can be used as a detector in analysis [46]. Therefore, the NMR analysis can provide valuable insight into enantiomeric resolutions by calculating binding constants based on chemical shift values of signals in the NMR spectra, providing information about the topology of the diastereomeric complexes. In the study [30], obtained values of the binding constant differed depending on the chosen signal in ^1^H spectra. Hence, the average apparent binding constants have been calculated to predict the HPLC outcomes. Based solely on the results of K values, the 3-acetoxy-2,2-dimethylcyclohexan-1-one isomers have been the most likely to be separated by HPLC analysis since α = 1.31. It has been determined that to predict chiral discrimination, α values should remain above 1.30 (when α is below 1.20, enantioselectivity would not be achieved). However, for the borderline values, the other parameters such as the enantiomeric discrimination of ^13^C NMR signal upon β-CD complexation and diffusion coefficients (HR-DOSY) of enantiomers could have an impact on the obtained results. Based on the tested examples, it is worth noting that both requirements should be additionally met to obtain reliable results and predict chiral selection in RP-HPLC, i.e., diffusion coefficients (above 60% of the complexed population) and large averaged ^13^C NMR signal splitting value (above 4–5 Hz) in the presence of chiral selector. The 3-acetoxy-2,2-dimethylcyclohexan-1-one has fulfilled all requirements leading to good chiral discrimination. Although for 3-hydroxy-2-methyl-2-(2-propynyl)-cyclohexan-1-one α = 1.25, which is a borderline value and the average ^13^C NMR signal splitting values were equal to 4.6 Hz, the value of complex population (p% = 65.6 ± 1.3) is responsible for the observed discrimination of its enantiomers in HPLC analysis. The order of enantiomers elution in HPLC has been correctly predicted by the obtained binding constants from the NMR part of the study.

A similar approach has been tested in the study of ethyl-phenylsulfoxide enantiomeric discrimination [38]. Even though the α value was found to be 1.22, which is in the borderline range and 62% of the complexed population, above the 60% threshold, the average ^13^C NMR signal chemical shifts splitting have been 2.7 Hz, suggesting that diastereomeric complexes are topologically similar. Utilizing complexation-induced chemical shifts in ^13^C NMR spectra enables more accurate prediction of RP-HPLC outcomes since it depends more strongly on the molecular geometry of complexes than ^1^H NMR signals that are largely influenced by the anisotropy. Based on those findings, it has been concluded that the HR-DOSY/^13^C NMR complexation-induced chemical shift methods lead to more accurate results than using ^1^H NMR chemical shift methods.

Nevertheless, the studies support the statement that NMR analysis can be applied for the prediction of chiral discrimination in reverse-phase HPLC.

A combination of NMR and molecular modeling studies have been performed to investigate the complexes of enantiomers of mandelic and hexahydromandelic acid [39]. The obtained α values indicate effective chiral recognition of enantiomers by application of native β-CD, namely 4.56 and 3.65 for mandelic and hexahydromandelic acid, respectively. Moreover, the free energy perturbation (FEP) approach correctly predicted the most stable model of diastereomeric complexes with tested isomers. It has been noticed that enantiomers can exist in two different orientations: A and B, in which the guest is located at the wider and narrower rim of the β-CD cavity, respectively. The enantiodifferentiation of hexahydromandelic acid is attributed to orientation A, whereas both orientations A and B contribute to the enantioselectivity of mandelic acid.

In the case of verapamil (VP), the binding constants have been calculated based on Scott’s plots constructed using the ^13^C NMR signals, to support CE analysis [42]. The average apparent binding constants’ values correlate well with the observation of chiral separation and the order of enantiomers in capillary electrophoresis, suggesting higher stability of (+)-VP-β-CD complexes compared to (-)-VP-β-CD.

In conclusion, NMR analysis can be successfully applied to propose the conformation of diastereomeric complexes and the penetration mode, based on changes in chemical shift in NMR spectra. Furthermore, the results from NMR can help to predict the outcomes of HPLC and CE analysis however, several factors described above should be fulfilled. The NMR results are often combined with molecular modeling since the comparison of experimental and calculated NMR properties is a good method for structure elucidation and verification. 

At this point, it should also be noted that the molecular modeling methods are also employed in the prediction of the applicability of β-CD as an auxiliary agent for enantioseparation. The applied approaches are usually similar, starting with the generation of the possible low energy structures of the host–guest complexes through molecular docking. The obtained structures are usually studied further using molecular dynamics (MD) simulations [109] and/or quantum mechanical (QM) calculations, both at the semi-empirical and density functional theory (DFT) levels [110].

### 6.4. Ultraviolet–Visible (UV–vis) and Fluorescence Spectroscopy (FS)

#### 6.4.1. Ultraviolet Analysis (UV–vis)

**Figure 6:**
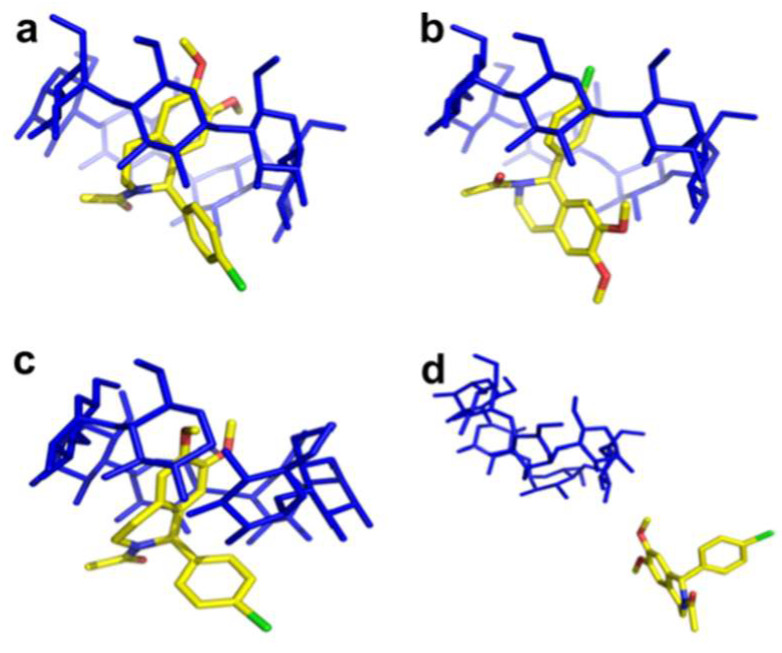
(R)-AA and (S)-AA complexed by β-CD (blue): (**a**) and (**b**) represent the results of docking studies for (R)-AA and (S)-AA, respectively; (**c**) and (**d**) show the average structures from MD simulations for (R)-AA and (S)-AA, respectively. Reprinted with permission from [90].

Although the differences between binding constant for complexes formed between each enantiomer and β-CD can be too minor to enable chiral recognition [50,54,83], there have been identified instances when only one of the enantiomers forms a stable complex with β-CD, like for AMPA receptor antagonist (AA), described in the following study [90]. The inclusion complexes of AA enantiomers and native β-CD have been investigated using UV–vis phase solubility diagrams and FTIR-ATR, supported with molecular modeling. The stability constants for complexes have been determined to be 15889 M^−1^ and 1079 M^−1^ for R-AA-β-CD and (R/S)-AA-β-CD complexes, respectively. The binding constant and solubility improvement for S-AA cannot be determined due to the lack of formation of stable complexes with β-CD. The results of the molecular modeling study have confirmed the significant differences in affinity between enantiomers towards β-CDs. As observed in molecular dynamic simulations, the R-AA has been constrained inside the cavity of CD during the entire simulation time; however, the S-AA has shifted rapidly away from the cavity (Figure 6). Interestingly, the R-enantiomer has been considered as a eutomer presenting higher AMPA antagonistic and anticonvulsant effects than the S-enantiomer [111].

#### 6.4.2. Fluorescence (FS)

The chirality of 1,1′-Binaphthol (BINOL) manifests due to hindered rotation around the bond between the naphthalene rings. The authors of the next work have tested the ability of native α- and β-CDs as chiral selectors in the case of BINOL enantiomers by performing FS analysis in the temperature range of 5–45 °C [70]. Based on the values of binding constants it has been concluded that both enantiomers display higher affinity to β-CD than α-CD; however, relatively small values of association constants resulted in poor enantioselectivity of CDs, attributed to the fact that BINOL molecules penetrate only slightly inside CDs cavities. Since β-CD has a larger cavity, both enantiomers of BINOL incorporate more deeply; however, the differences are minor. It has been observed that the driving force of the formation of the stable inclusion complexes can be attributed to non-bonded van der Waals interactions The findings have been established by thermodynamic properties, and the results form molecular modeling calculations. The poor enantioselectivity and low values of association constant can be explained by the BINOL aggregation in aqueous solvents, and therefore, only a small portion of the guest forms the complexes with CDs. Due to the high level of uncertainty of the calculated binding constant, it could not be concluded which enantiomer creates more stable complexes. Additionally, the enantioseparation of BINOL has been investigated in a different study [74], and the results have exposed that S-BINOL has a higher affinity toward β-CD, with the binding constants of 245 ± 4 and 176 ± 25 M^−1^, for S- and R-enantiomers, respectively.

**Figure 7:**
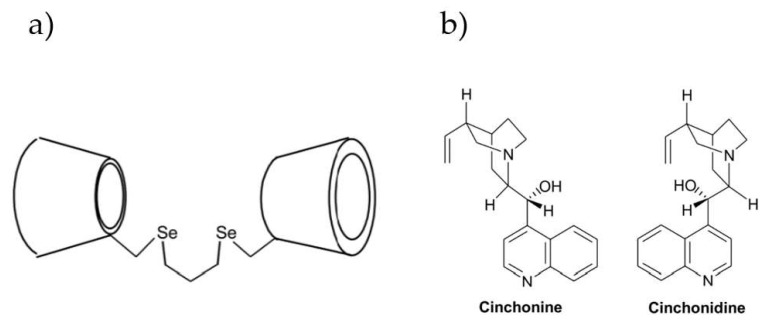
(**a**) the schematic structure of dimeric 6,6′-trimethylenediseleno-bridged, bis(β-CD)s, (**b**) the skeletal formula of cinchonine and cinchonidine Reprinted with permission from [72].

Since some guest molecules, like cinchonidine and cinchonine (the skeletal formula of enantiomers presented in Figure 7b), display intense fluorescence in aqueous solutions and are responsive to changes in the environment, the fluorescence titration method has been applied to investigate their inclusion complexes with β-CDs and to assess the selective binding affinity [72]. The work was augmented also by recording the 2D-NMR spectra to provide a deeper understanding of the binding model. Based on the results, it has been observed that both cinchonidine and cinchonine only partially fit into the β-CD cavity, due to the large size of the guest molecules. Hence, the binding constants determined for those chinchona alkaloids were similar. However, the application of dimeric β-CD significantly improves the binding strength between host and guest molecules, leading to an improvement in the ability of cyclodextrin as a chiral selector by increasing the α ratio from 1.08 to 1.96 for native β-CD and dimeric 6,6′-trimethylenediseleno-bridged bis(β-CD) (schematic stricture(β-CD)s presented in Figure 7), respectively, through cooperative guest molecule binding by two adjacent cavities. This example has shown that the incorporated molecule’s size can affect CDs’ ability as chiral selectors.

#### 6.4.3. Isothermal Titration Calorimetry (ITC)

The author of the work [83] has performed the ITC analysis to identify the key thermodynamic factors affecting the chiral recognition process. The relationship between the guest’s structure and the thermodynamic parameters has been discussed. It has been noticed that the mode of penetration, the size of the guest, and whether the center of chirality is inserted into a cavity of cyclodextrin can play a significant role in the enantioselectivity. The results for relatively simple guests like phenylbutyric acid derivatives have shown that chiral recognition is most likely achieved when the distance between the asymmetric center and the hydrophilic group of a guest is as large as possible, due to the influence of the penetration mode of the guest molecule into the cavity. Therefore, the enantioselectivity has been observed for 3-phenylbutyric acid enantiomers, whereas no discrimination has been obtained for 2-phenylbutyric acid isomers. Good chiral recognition has been detected for N,N-dimethyl-1-ferrocenylethylamine due to restricted movements within the cavity, leading to α value of 1.2, and the enantioselectivity has been described as being governed by entropic contribution. The chiral guest that possesses more rigid penetrating groups is more likely to show chiral recognition compared to those with more flexible groups, as the latter can adapt their conformation inside the β-CD cavity like in the case of 1-cyclohexylethylamine, in which no chiral discrimination has been detected. Those aforementioned findings for native β-CD have been preserved with the application of cationic 6-amino-6-deoxy-β-CD (am-β-CD) in the analogous study [88], leading to the conclusion of the possibility of a “chiral template” between native β-CD and its derivatives.

#### 6.4.4. Other Methods

In the next work [89], the authors applied phase solubility methods to assess the binding constant differences of complexes formed between the stereoisomers of ibuprofen and CDs. The technique involves adding a fixed amount of drug to an unbuffered aqueous solution of β-CD, exceeding its solubility and then, based on UV–vis absorbance, calculate the binding constants of complex formation. The results indicated the increase of solubility of ibuprofen through complexation to β-CDs and that no significant chiral solubility discrimination has been observed in the case of native β-CD complexes. Due to similar values of binding constants for R- and S-enantiomers, the α ratio was equal to 1.03.

In the next study, the chiral separation system has been created by covalently binding β-CD onto the surface of cellulose membranes [96]. The effectiveness of this approach has been tested to separate a racemic mixture of tryptophan (Trp). It has been revealed that L-Trp exhibits higher affinity to β-CD, manifesting with a higher value of the binding constant of 0.043 mM^−1^ compared to D-Trp with a binding constant of 0.031 mM^−1^. This finding is in line with the previously mentioned study [69]; however, obtained α values differ significantly between both studies, i.e., 2.63 and 1.39 by using fluorescence [69] and ultrafiltration method [96], respectively. Despite the relatively low separation factor by employing an ultrafiltration approach, a multi-stage cascade in the membrane process can be utilized to meet the demands of the pharmaceutical industry requirements. Hence, it provides the opportunity to distinguish between two tryptophan enantiomers by utilizing the distinct binding constants of the enantiomers with β-CD.

In the next study [91], the solubility and thermodynamic properties of praziquantel (PZQ) and its enantiomers have been analyzed, and the binding constants have been obtained from the phase solubility diagram. Due to the overlap in slopes for the enantiomers in their 95% confidence intervals, the estimated K values have not differed significantly. The X-ray and IR-analysis supported those observations, as no significant differences between the complexes with each isomer have been noted. Interestingly, the ability to form the complexes with β-CD for the isolated stereoisomers has been reduced since the stability constant for racemic PZQ has been higher than for (-)-PZQ, namely 368 M^−1^ and 335 M^−1^, respectively. The stability constant for both (±)-PZQ and the isolated isomers indicates a moderate level of affinity which may be attributed to the rigidity of the guest molecule and its inability to be completely incorporated into the β-CD cavity, at least within the tested range of molar ratios.

### 6.5. Comparison of Analytical Methods within the Single Study

As described in Section 5.2, multiple analytical methods can be used to determine the complex stability constants. It could be expected though, that the obtained results on enantioselectivity should be similar, regardless of the applied analytical method. However, as the complex formation and stability are very prone to changes upon the variation in temperature, viscosity, or presence of other molecules in the system, unfortunately, the results can vary significantly upon the choice of a particular method. Therefore, in some of the reviewed works, more than one approach has been applied to obtain the binding constants. Such extra work has been usually justified by the authors as resulting from a demand to clearly assess the effectiveness of β-CD as a chiral selector, regardless of the applied analytical method.

The comparison of two analytical methods, i.e., HPLC and CE, to achieve chiral selection of basic drugs has been performed [49]. According to the results for brompheniramine and cyclopentolate, the application of both methods leads to enantioselectivity, with the application of native β-CD as a chiral selector. It is worth noting that although the K values have been determined at different pH, 2.5 and 7.0 for CE and HPLC, respectively, the α ratios obtained using different approaches were comparable. For both techniques, it has been observed that (+)-brompheniramine formed more stable complexes than its (-)-isomer. In the case of cyclopentolate, the K values determined using CE have been 5 times higher than those obtained using HPLC. The authors of the reviewed work have explained the observed differences in values as resulting from the opposite mobilities of the chiral selector and analyte. When the mobility of the chiral selector is opposite to the mobility of the analyte, even a small difference in complexation between enantiomers can produce good enantioselectivity. The variations in stability constant estimation can result from the impact of the solvent on complexation, such as the introduction of 20% ethanol to the mobile phase in the HPLC experiment. Nevertheless, both methods can be used together to examine complexation phenomena of native β-CD.

The study on chosen antifungals has shown that higher chiral recognition does not always correlate with the highest strength of interaction between analyte and CDs [54]. While the complexes formed between econazole and sulconazole with β-CD have been characterized by the higher values of apparent binding constants, compared to HP-β-CD, the better enantiomeric resolution has been obtained using the latter. In the case of ketoconazole, the chiral recognition has not been observed using native β-CD, even though the obtained binding constants have been an order of magnitude larger than for TM-β-CDs, which was found to be successful in enantioseparation. The finding demonstrates that the relative ratio of binding constants between complexes with each enantiomer is more crucial than the absolute value of the association constant and the strength of interactions. The chiral discrimination of bifonazole, econazole, sulconazole, and miconazole enantiomers have been also tested using RPLC analysis [75]. Surprisingly, there were some discrepancies between both studies, even though both analyses have been performed precisely at pH = 3.0. Regarding bifonazole, in CE analysis [54], no chiral discrimination has been observed at a temperature of 15 °C and with the concentration of β-CD 1–15 mM (α = 1.00). While using RPLC, the enantioselectivity for bifonazole enantiomers has been obtained at both 5 and 20 °C, as indicated by α values equal to 1.5 and 1.38, respectively [75]. The obtained K values for most tested compounds differ significantly by the order of magnitude between both studies, and the smallest differences have been noted for econazole, for which the values were in the range 168-229 M^−1^ [75] and 622-737 M^−1^ [54], measured at 20 °C and 15 °C, respectively. Unfortunately, the order of eluted enantiomers in CE analysis [54] has not been clarified, making it impossible to compare between studies.

In the next work [40], the authors investigated the reverse migration order of enantiomers of antihistaminic drug brompheniramine (BrPh) in CE analysis when either native β-CD or TM-β-CD were used. The binding constants calculated from chemical shift differences observed in ^13^C NMR spectra revealed that (+)-BrPh has a higher affinity to native β-CD than (-)-isomer. Although the α ratio is relatively low, namely 1.03, the particular interactions and selectivity of native β-CD make it an effective chiral selector in the case of BrPh, and the NMR findings have been in line with the order of enantiomers in CE analysis. It is worth mentioning that binding constants have been calculated assuming the 1:1 stoichiometry, whereas the X-ray analysis has shown that a 1:2 complex is formed in the case of native β-CD. However, it may be necessary to take into account potential disparities between the structure in a solution and a solid state.

## 7. Key Factors Influencing the Effectiveness of Enantioselectivity of Β-CD

Several factors should be thoughtfully chosen to obtain effective chiral separation using β-CD as a chiral selector. In this part of the review, the examples in which the authors have tested various conditions of analysis will be discussed. The works have been divided regarding tested factors; however, in many cases, the optimization of more than one factor has been performed in the studies simultaneously.

### 7.1. Additives

In the next study [78], the authors investigated the enantioseparation of cyclopentolate (CP), 2,6-diketopiperazine derivative (2,6-DKP) and methylphenobarbital (MPB) using native β-CD as a component of the mobile phase in HPLC, either with or without the addition of ion interaction reagents (IIR). A significant increase in obtained values of binding constants has resulted from the addition of IIR, which could possibly lead to enhancement of enantioseparation (Table 3). However, for 2,6-DKP and MPB, with the increasing length of the alkyl chain IIR, the loss of enantioselectivity was observed, whereas for CP, the positive effect on retention and chiral recognition was noted, most probably due to its higher basicity compared to other compounds. The decrease in enantioselectivity with the longest alkyl chain IIR can be associated with the easy incorporation of IIR in the cavity of CD, thus with significant replacement of the analyte from the cavity of CD. Furthermore, the study emphasized the effect of temperature on retention rates. It was observed that for the mobile phase containing sodium 1-butanesulfonate (C4) with β-CD in the temperature range of 15–25 °C, the process of complexation dominated; however, from 25 to 40 °C, the interaction with the stationary phase overcame the complexation. For the mobile phase with C18 and C12, the interaction with the stationary phase dominated in all studied temperatures, resulting in a decrease in K values.

In conclusion, the presented results indicated that the application of IIR can either increase or decrease the effectiveness of chiral discrimination, depending on the nature of the analyte (base, acid, and amphoteric), temperature, and the length of a chain of IIR. Nevertheless, the IIR additive to the mobile phase can be a promising method of enhancing enantioseparation in HPLC.

**Figure 8:**
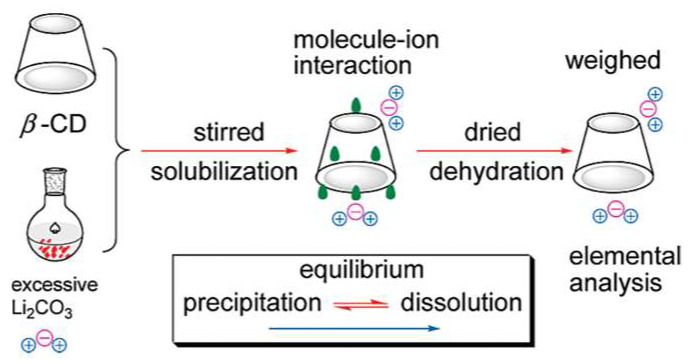
Schematic diagram illustrating the solubilization of Li_2_CO_3_ by β-CD. Reprinted with permission from [69]. Copyright 2009 American Chemical Society.

As has been mentioned before, in some cases the addition of a third component can result in better chiral recognition [66,67]. However, there have been known cases that the formation of ternary complexes can lead to a decrease in chiral recognition such as for tryptophan (Trp) [69]. The authors have investigated the pH and salt effect on intermolecular interactions between Trp and β-CD and therefore changes in binding affinity and chiral recognition behavior of the host. In the presence of Li_2_CO_3_ at the same pH beyond the increase in binding strength resulting in higher binding constants for D- and L-Trp, the negative influence on the recognition behavior has been observed. For instance, at pH = 10.4, the α ratio slightly decreases from 2.63 to 2.34. In the absence of Li_2_CO_3_, at pH = 11.0, besides observed stronger binding affinity and higher values of K, the ability of chiral recognition of β-CDs decreases at higher pH, and the α ratio changes from 2.34 to 2.15. The molecule–ion interactions between Li_2_CO_3_ and β-CD have been crucial for increasing the strength of the bond between CDs and Trp, simultaneously influencing the selectivity of CDs towards D-and L-Trp. The schematic diagram of the solubilization of Li_2_CO_3_ by β-CD has been presented in Figure 8. The presence of Li_2_CO_3_ led to a more polar environment, which promoted the inclusion of the indole ring of Trp within the cavity of the CD. Furthermore, Li^+^ cations preferred to coordinate with hydroxyl groups, reducing the polarity of the cavity. At higher pH, the presence of Trp in an anionic form enhanced the formation of intramolecular hydrogen bonds in the Trp molecule, forming more stiff structures and affecting the differences in chiral carbon atoms between D- and L-Trp, potentially explaining the lower chiral recognition of cyclodextrin at higher pH. Additionally, the authors have performed a series of analyses for selected carbonates and lithium salts to estimate which ion group may have a greater impact on the spectral differences between enantiomers. Although the effect of Li^+^ on recognition behavior should not be disregarded, it has been concluded that CO_3_^2−^ ions play a more significant role in this process.

### 7.2. Temperature and pH

The authors of the next work investigated the impact of temperature and pH on a chiral selection of α-cyclohexylmandelic acid (CHMA) by using native β-CD and Cu_2_(II)-β-CD for chiral high-speed counter-current chromatography (HSCCC) [92]. It was observed that in the tested temperature range (283.15 K–308.15 K), the increase in temperature led to a decrease in the distribution ratios of the CHMA enantiomers in two-phase solvent system analysis, and consequently decrease in separation factors. Despite the observed decrease in K and α values due to weaker interactions between the analyte and chiral selectors, the underlying mechanism of chiral recognition has become intact, allowing the enantioseparation within the tested temperature range. For both selectors, the enantioseparation factor first reached a plateau and then decreased sharply with the increase in pH value within the tested range. However, Cu_2_(II)-β-CD is stable only in alkaline solutions. Hence, for further analysis, the pH values of the aqueous phase equal to 9.81 and 3.51 were chosen for Cu_2_(II)-β-CD and β-CD, respectively. The results revealed that in comparison to native β-CD, the application of Cu_2_(II)-β-CD as a chiral selector significantly improved the effectiveness of enantioseparation of CHMA enantiomers mostly due to the formation of electrostatic interactions between Cu_2_(II)-β-CD and the deprotonated carboxyl group of CHMA.

#### 7.2.1. Temperature and Thermodynamic Properties

The effect of temperature and composition of the mobile phase on retention and stereoselectivity has been investigated for chiral stationary phase systems in HPLC analysis [79]. It has been observed that an increase in temperature leads to the reduction in resolution, selectivity. and retention factors.

Further, the impact of temperature and β-CD concentration in the mobile phase using reverse-phase liquid chromatography has been tested on the imidazole derivatives, employed for the treatment of onychomycosis [75]. It has been noted that the values of binding constants for all tested compounds decrease with the increase in temperature, and above 50 °C no enantioseparation has been observed for all tested imidazole derivatives. The bifonazole, econazole, sulconazole, and miconazole enantiomers have been successfully separated in the temperature range from −8 to 40 °C, with the concentration of β-CD equal to 1, 1.5, 2, 2.5, and 3 mM. Additionally, in all cases, the S-isomer eluted before the R-enantiomer. However, the resolution was strongly influenced by the applied column temperature. For concentrations of β-CD below 0.5 mM, the enantiomers were not separated at any of the studied temperatures, due to the shift of the equilibrium of complexation towards the free solute, resulting from the low concentration of the chiral selector in the mobile phase. At the β-CD concentration below ca. 2.25 mM, chiral recognition is enthalpy-driven with the favor of the formation of stereoselective hydrogen bonding between guest and hydroxyl group of β-CD or steric interaction. On the contrary, at a concentration above 2.25 mM, the stereoselectivity occurs mainly due to the formation of van der Waals interaction and hydrogen bonds with the cavity of β-CD.

The effect of temperature on binding constants has been examined for the complexes of carvone (Crv) enantiomers with native β-CD, using NMR [41]. At room temperature, the apparent binding constant of the S-enantiomer has been twice as high as R-carvone, while at 45 °C, both enantiomers have association constants that are two orders of magnitude smaller, and the S-isomer inclusion is slightly weaker compared to the R-Crv. With an increase in temperature, the inclusion systems undergo dissociation resulting from the breaking of hydrogen bond, and at 318 K, they become significantly weaker compared to room temperature. The quantum and semi-empirical calculations exposed the most stable model for the S-Crv-β-CD complex, which is supported by molecular mechanics and experimental results, indicating a preferential inclusion of the S-Crv enantiomer to β-CD cavity. Moreover, based on quantum mechanical calculations, the importance of multiple hydrogen bonding interactions for the stability of the S-Crv-β-CD complex has been highlighted, since the R-enantiomer is not likely to form host–guest hydrogen bonding interaction.

In the next work [31], the authors investigated the chiral recognition of a racemic mixture of flavanones by β-CD and its derivates through NMR experiments. Binding constants and thermodynamic parameters were determined from the analysis of the splitting of the signals in ^1^H NMR spectra. The chiral selection was achieved for 2-phenyl-2,3-dihydro-4H-chromen-4-onen (FL) by application of all tested CDs, whereas for (2-(2-hydroxyphenyl)-2,3-dihydro-4H-chromen-4-one (2’OHFL), application of native β-CD for that purpose was unsuccessful. The results for 2-(4-hydroxyphenyl)-2,3-dihydro-4H-chromen-4-one (4’OHFL) have been taken from the authors’ previous work [32]; however, they were analyzed in comparison to FL and 2’OHFL in study [31]. Although the obtained K values for all formed diastereomeric complexes decreased with the increase in temperature, the α ratio behaved the opposite: at the higher temperature, the binding selectivity was higher. It would suggest that at higher temperatures, better chiral recognition should be presented. However, in NMR spectroscopy, the enantiomeric differences in spectra depend on the magnitude of the K values. Consequently, in higher temperatures, the chemical shifts for both enantiomers were more similar. Therefore, better enantiomeric differentiation has been observed at lower temperatures, suggesting that large α value does not always indicate more effective chiral recognition.

Afterward, the authors performed the thermodynamic analysis to understand the contribution of each component to chiral recognition. ΔG values for all studied complexes were negative, which implies the spontaneous formation of complexes between studied flavanones and CDs. The formation of 4’OHFL/β-CD complexes turned out to be enthalpy-favored, with van der Waals interaction and hydrogen bonds being the main contribution to complex formation. In the case of FL and native β-CDs complexes, the process of chiral selection has been determined to be entropy-driven. Additionally, the obtained lower values of ΔH and ΔS for FL-β-CD in comparison to other complexes could suggest that the formation of this complex required the expulsion of more water molecules from the cavity than for the formation of other complexes.

#### 7.2.2. pH Value

The type and strength of interaction between guests and β-CD can depend on the form in which the guest exists, i.e., acidic, basic, or neutral. Therefore, pKa values should be taken into account to properly choose the optimum pH values applied in the analysis. The importance of pH in the case of the formation of diastereomeric complexes has been highlighted, based on the examples provided in different parts of this review.

In the example of racemic metomidate (MET) [29], the effectiveness of NMR analysis to predict results in CE has been tested. Detailed investigations including the application of several chiral selectors, pH, and the ionic strength of the buffer on chiral recognition have been performed. Contrary to using α and γ-CD, a substantial effect of native β-CD on the ^1^H NMR spectra of (±)-MET has been observed. The splitting and downfield shift of particular signals have shown that the β-CD is a more effective chiral selector than the other tested native CDs. The differences in NMR spectra allowed to calculate apparent binding constants, suggesting different stability of diastereomeric complexes, supporting the occurrence of chiral recognition. A good correlation between NMR measurements at different pH and corresponding CE analysis at the same pH has been detected. The condition of analysis has a crucial impact on the appearance of enantioseparation. For instance, no or very poor chiral selection has been observed at pH equal to 2.4 and 6.0 in the CE system, whereas it has been achieved at pH = 3.5, correlating accurately with the signal splitting in NMR spectra. It is worth noting that both the complexation ability of chiral selector and electrophoretic mobilities are pH-dependent, and both components can affect the outcome of CE analysis. Furthermore, the increase in the buffer concentration may enhance the ionic strength that can likewise impact the interactions between the chiral selector and solute as well as the electrophoretic mobilities. Hence, the increase in the ionic strength and pH value can influence ultimately peak resolution and chiral selection using β-CD.

In the case of naproxen (Nap), it has been reported that in acidic pH, below pKa value, Nap exists as a neutral molecule and hence can form hydrophobic interactions and hydrogen bonding between hydrogen in a carboxylic group of Nap with a hydroxyl group of CD [66]. However, at pH above 6, it was observed that room temperature phosphorescence (RTP) emission almost disappeared and Nap exists as an anion, leading to a reduction in binding affinity to β-CD and worse chiral recognition.

**Figure 9:**
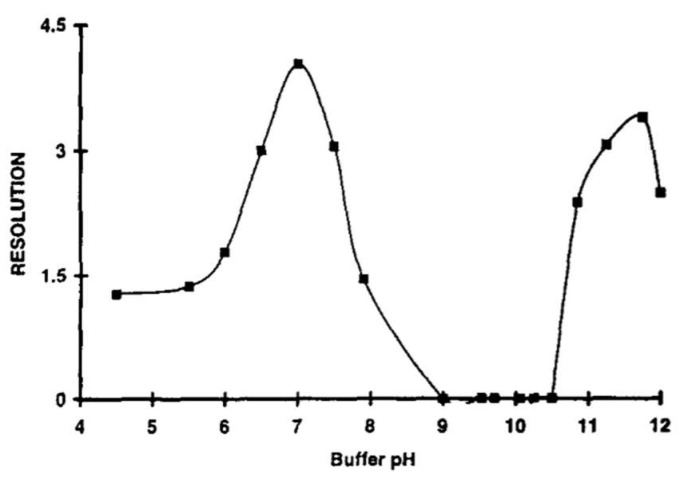
Variation in the resolution with electrolyte pH during 5-OH-DPAC enantioseparation. Fused-silica capillary column, 70 cm × 50/μm I.D.; applied voltage, +18 kV; buffer 50 mM phosphate-borate-6 mM β-CD-8 M urea; detection at: 210 nm; temperature, 25 °C. Analytes were injected on-column using hydrodynamic injection for 1s. Reprinted with permission from [58].

Different pH values can lead to changes in the charge of molecules, hence to the different outcomes of the analysis. Such a case has been observed in the example of 3,4-dihydro-2H-1-benzopyran (DPAC) derivatives, studied by capillary zone electrophoresis (CZE) [58], in which tested compounds are tertiary amines for which the ionization constants are placed in the 5–8 pKa range. The impact of applied pH values on the resolution of 5-OH-DPAC enantiomers has been tested using the buffer pH range of 4.5–12. As shown in Figure 9, the highest resolutions have been achieved at pH values equal to 7 and 11.75. In addition, at pH = 7, baseline separation has been accomplished in less than 15 min. The pH value of 7.0 has been chosen to partially protonate solutes, allowing cationic species to move toward the cathode with the velocity of electrophoretic migration plus that of electroosmotic flow. At the range 9.0–9.7 no enantioseparation has been detected due to the neutral form of the solute. However, at the pH value above 10, at which enantiomers are in their anionic form, the reverse order of peaks has been observed, and the first peak has been assigned as the S-enantiomer, the second to the R-isomer. Furthermore, other factors like ionic strength and concentration of β-CD have been investigated in this paper. The experimental value of β-CD optimal concentration which gives the highest enantioselectivity has been in agreement with theoretical calculations.

The optimization of the key factors such as pH range, buffer composition, and buffer and chiral selector concentration, and the determination of optimal conditions have allowed the successful enantioseparation by β-CD in the capillary zone electrophoresis system.

### 7.3. Solvent

The effects of the pH and applied solvent have been also tested on the mianserin, trimipramine, and thioridazine enantiomers in CE analysis [57]. It has been concluded that the obtained binding constants tend to decrease with the increase in the pH. It could be deduced that the decrease in the polarity of the solvent resulted in a significant decrease in binding constant, from around 10^4^ in water to 10^1^ in formamide and around 10^−2^ in N,N-dimethylformamide (DMF). In a nonaqueous environment, there is a lack of hydrophobic interactions, which play a significant role in the formation of the stable complexes, leading to the lower K values. Consequently, the optimum concentration of β-CD in aqueous solution is in the high micromolar range, whereas in formamide, the required concentration is much higher, around 100 mM. It has been observed that in aqueous media, developing an effective method to separate tested compounds is challenging due to very low concentrations and rapid enantioselectivity changes. The study has revealed that even at non-optimum concentrations of the chiral selector, other experimental parameters like ionic strength and the addition of tetraalkylammonium (TAA^+^), short-chain cationic surfactant, also play a significant role in CE analysis. The effect of the temperature has been also tested at the range of 25–40 °C. With the decrease in temperature, better separation was achieved due to the increase in the viscosity of the medium, thus increasing the migration time. The results have shown the corresponding trend in nonaqueous environments as in aqueous media.

The addition of methanol can lead to a decrease in the binding constant due to competition between methanol and the tested compound-guest [35]. Furthermore, in some cases, the presence of methanol can improve chiral recognition [55,64]; however, there are known examples where a decrease in both binding affinity and chiral discrimination has been observed [80]. However, in the case of tioconazole [61], although increasing the methanol concentration resulted in a significant decrease in the K values from about 1.6 × 10^3^ to 0.24 × 10^3^ M^−1^, α ratios remained at a similar level of ca. 1.2 at the concentration in the range of 0–25%.

### 7.4. Optimal Conditions

Since more than one key factor such as pH, the concentration of chiral selector, temperature, presence of additives, and diameter of the capillary can influence the enantioselectivity in capillary electrophoresis, the optimal conditions have been determined for 10 selected chiral drugs with β-CD as chiral selector [55]. It has been noted that the application of a capillary of 20 μm diameter resulted in notable advantages including resolution improvement and faster analysis time compared to a capillary with a diameter of 50 μm. Optimization of conditions for enantiodiscrimination would likely require lower concentration of chiral selector, which is especially important when native β-CD is being employed due to its limited solubility in aqueous solutions (solubility: 18.5 mg/mL, 25 °C [112]). For the CE analysis, the buffer pH of 2.5 has been used for the enantioseparation of 10 basic drugs allowing to boost the intermolecular forces between β-CD and analyte. Those interactions involved the electrostatic forces, hydrogen bonds, van der Waals forces, and dipole–dipole interactions. An increase in the strength of hydrogen bonding between the polar functional groups of the β-CD and the analyte and protonation of the basic amino groups of the guest can lead to improved electrophoretic migration and increased effective separation, due to the formation of the more stable CD-analyte complexes [55]. The optimal conditions for achieving enantioseparation for a particular drug are summarized in Table 4.

It has also been noted that an increase in temperature leads to a decrease in migration time and resolution (R_s_). Therefore, the temperature was maintained at 15 °C, and the temperature was increased up to 30 °C, to allow more rapid separation only when the R_s_ was high enough (>1.5) to achieve enantioseparation.

Since the higher concentration of buffer can decrease the electroosmotic flow (EOF) and increase the separation time due to an increase in ionic strength, the higher concentration of buffer has been applied only when the obtained resolution has been lower than 1.5. However, the solubility of β-CD has been enhanced by varying the concentration of urea in the background electrolyte. Higher concentrations of urea can cause an increase in the viscosity which can affect the mobility of solutes.

It is worth mentioning that the addition of organic solvent can negatively affect the stability of analyte-β-CD complexes due to competition between additives and analyte. Alternatively, the addition of organic solvent can improve the chiral recognition due to its differential impact on the stability of complexes formed for two enantiomers with β-CD. For instance, it has been observed that the application of 20% methanol to propranolol increased its R_s_ to 3. Additionally, the addition of triethylamine (TEA) can enhance the chiral recognition, and adding TEA to isoproterenol has resulted in an improvement in the R_s_, allowing for separation at 20 °C, whereas the addition of TEA to alprenolol resulted in a longer time of separation (ca. 70 min) without any significant impact on R_s_.

Effective chiral recognition can be influenced by the concentration of the chiral selector. Hence, the resolution mechanisms depend on the formation of inclusion complexes and particularly those complexes formed mainly by hydrogen bonding; complexes with compounds of two ortho-hydroxyl groups in the aromatic ring are less preferred than meta or para position due to steric hindrance. Therefore, it has been observed that isoproterenol requires a higher concentration of β-CD to achieve enantioseparation compared to metaproterenol and terbutaline.

**Figure 10:**
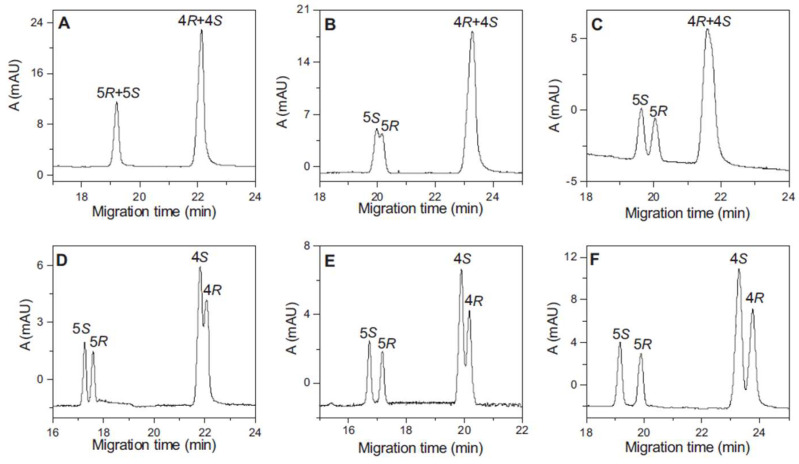
CE analysis (**A**) and ACE separations of (R,S)-enantiomers of ANPs 4 and 5 (**B**–**F**) at increasing concentrations of the chiral selector β-CD. (**A**) 0 mM; (**B**) 5 mM; (**C**) 10 mM; (**D**) 15 mM; (**E**) 20 mM; (**F**) 25 mM. Reprinted with permission from [52].

The effect of the concentration of β-CD has been tested as well as for acyclic nucleoside phosphonates (ANPs) [52]. The increase in concentration of the chiral selector significantly enhanced its effectiveness, allowing enantioseparation in CE (see Figure 10).

Even slight structural differences in structurally related compounds can hinder enantiomeric separation, making it challenging to predict optimal conditions. Understanding these similarities, based on results from analysis at optimal conditions, can aid in further studying the enantioseparation of β-CD for similar drugs using CE analysis.

### 7.5. Comparision with Chosen Other Chiral Selectors

Although this review is focused on the application of native β-CD as a chiral selector, in several papers, derivatives of or native α-, γ-CD have been also analyzed and compared to β-CD.

In the chiral recognition process, the size of the cavity can play a significant role like in the case of 1,1′-binaphthyl-2,2′-diyl hydrogenphosphate (BNP) [71]. The native α, β, and γ-CDs have been tested as chiral selectors using FS measurement. The higher enantioselectivity has been observed by applying β-CDs due to constraints of movement of BNP inside the cavity of β-CD and precise conformational complementarity. The native α-CD is less likely to form stable diastereomeric complexes with BNP due to the small size of the cavity (see Figure 1), whereas the reduction of chiral discrimination has been observed for γ-CD due to the larger cavity enabling the greater mobility and freedom of conformational changes within the cavity. Similarly, no chiral recognition has been observed for ferrocenylethanol enantiomers with α- and γ-CD as a mobile phase additive, while successful separation has been achieved with β-CD in microcolumn liquid chromatography [82]. Further considering the signs of the components of Gibbs free energy, the chiral recognition process of BNP using β-CDs has been determined to be enthalpy- and entropy-driven at low and high concentrations of β-CD, respectively [71]. Unexpectedly, for the S-enantiomer, the 1:1 stoichiometry has been indicated at the range of higher concentrations of β-CD, while at lower concentrations, the formation of both first- and second-order complexes has been detected. These findings resulted in the possibility of two BNP molecules complexing with one molecule of β-CD, creating 2:1 complexes. At higher concentrations of β-CD, the association constants have been determined to be 230.2 M^−1^ and 369.6 M^−1^, for R- and S-BNP complexes, respectively. The results aligned with the anisotropy measurements, which indicate a higher amount of anisotropy for the S-BNP complex and expose preferential inclusion of S-BNP to β-CD. The stronger interaction of S-BNP with β-CD compared to antipode enantiomer and good chiral recognition have been also observed in the work [74].

Another study analyzed the interactions between zileuton, a chiral 5-lipoxygenase inhibitor, and β- and γ-cyclodextrins using solubility measurements, ^1^H NMR, UV, and circular dichroism (CD) spectroscopies [44]. The obtained results were consistent with each other and indicated a stronger affinity of zileuton to β-CD in comparison to γ-CD. However, the K values with standard deviation have been practically indistinguishable for diastereomeric complexes of zileuton with β-CD, whereas in the case of γ-CD, showed a slight preference for (+)-enantiomer, which was in agreement with observations in NMR, UV and CD spectra.

In the case of noradrenaline, while native β-CD has been found to have higher binding constants compared to carboxymethyl-β-CD (CM-β-CD), CM-β-CD has a larger binding selectivity, which enables enantiodiscrimination [36]. Similarly, the enantiomers of dimethindene (DIM) have shown a significantly higher affinity for native enantiomers than for heptakis(2,3,6-tri-O-methyl-)-β-CD (TM-β-CD), represented by higher values of binding constant 77 and 17 M^−1^ compared to 457 and 504 M^−1^, for S- and R-DIM for TM-β-CD and native β-CD, respectively [37].

The authors of the next work investigated the interactions between selected drugs and cyclodextrins [53]. Although native as well as derivates of CDs were tested in CE, binding constants were obtained at pH 2.5 and 7.0 only for complexes with 4 representative drugs: paliperidone, propranolol, risperidone, and verapamil, since it would be too time-consuming to determine the binding constant for all created complexes. Both native β-CD and poly-β-CD were chosen as chiral selectors since they have shown good complexation abilities. The polycyclodextrins are synthetized through the direct copolymerization of CD molecules with various monomers such as epoxides, carboxylic acids or acrylamides serving as cross-linkers [113]. In the case of the reviewed paper, citric acid has been used as cross-linking agent [53]. Based on the results presented in Table 5, the application of polymeric form resulted in a 65% to 517% increase in apparent binding constants, which led to an improvement of enantioseparation of the tested compounds. The increase in binding constant determined for poly-CDs can be related to the presence of additional steric constraints causing the polymeric network to favor the inclusion of guests in the cavity of CD. It is worth mentioning that the impact of pH on the complexation has been significant, generally resulting in the higher value of K obtained at 7.0 compared to pH equal to 2.5, since all four examined compounds at both conditions are cationic. In the case of poly-CDs, the higher K values have been explained by the formation of ionic interactions between cationic compounds and ionic forms of poly-CDs at neutral pH due to deprotonation of citrate crosslinks. Consequently, the poly-cyclodextrins have shown high enantioselectivity towards tested drugs and could be used as effective chiral selectors, particularly in capillary electrophoresis.

Consequently, the differences between K values of the enantiomers are more important for accurate assessment of chiral selection than the absolute values of binding constants, and the appropriate chiral selector should be chosen depending on the size and conformation of a guest molecule as well as the physicochemical properties of the compound which manifest in various types of guest–host interactions.

### 7.6. Stoichiometry of the Complexes

Using gas–liquid chromatography, the stoichiometry of created complexes can determine whether enantioselectivity can be achieved in a particular case [95]. It has been observed that for monoterpenes, the enantioseparation by using native β-CD is attributed to the second step of complexation through the formation of 1:2 complexes, and for 1:1 stoichiometric complexes, the chiral selection has not been observed. Interestingly, the stability order of the complexes formed between β-CD with fenchone enantiomers was reversed upon the addition of β-CD to the mobile phase. Initially, in the absence of a chiral selector in solution, (+)-isomer created stronger complexes than antipode, whilst when the column was coated with β-CD and β-CD was added to the mobile phase simultaneously, the (-)-isomer had a higher affinity towards β-CD, leading to reverse the stability order. As it has been shown for monoterpenoids, the stoichiometry of created complexes can impact the effectiveness of chiral selection. Therefore, the application of a chiral stationary phase in conjugation with a chiral selector added directly to the solution can enhance the enantioselectivity. However, this approach requires the formation of stable 1:2 complexes between tested compounds. The molecular modeling methods can aid the evaluation of whether the formation of complexes is spontaneous and if the structures of those complexes differ significantly between enantiomers to allow the chiral selection.

### 7.7. Similarities and Discrepancies between Studies

In the reviewed works we have found several compounds that have been investigated in multiple studies, which allowed the critical comparison of the outcomes. While in some cases the results align with each other and provide similar conclusions, it should be noted that in many works, the inconsistency between the published data exists. Furthermore, those discrepancies have been observed even when the same method for the determination of K values even under very similar conditions has been used. In this paragraph, the examples of such compounds investigated by different researchers have been gathered and compared.

#### 7.7.1. Ketoprofen

In the work [65], the authors investigated an application of β-CD as chiral selectors for ketoprofen (KP) enantiomers, using several spectroscopic techniques. The FTIR and Raman spectra of β-CD and ketoprofen mixture significantly differ from those obtained for the isolated β-CD and isomers in characteristic band positions and their intensities, revealing the complex formation. In addition, the observed differences in Raman spectra for R- and S-enantiomers complexes have shown that Raman spectroscopy can be applied for chiral analysis for ketoprofen enantiomers. The UV and FS spectra have been utilized to quantitively assess the complex formation and to determine the stoichiometry of complexes. It is noteworthy that unionized enantiomers interact similarly with β-CD, therefore enantioselectivity was recognized only in neutral and not in acidic forms. Based on the results, R-ketoprofen has a greater binding constant (K) than S-ketoprofen, suggesting that β-CD forms inclusion 1:1 complexes more preferentially with R-enantiomer. The negative value of Gibbs free energy (ΔG) at 303 K for both enantiomers supports the observation of a spontaneous formation of complexes with β-CD, however, with the preference for R-enantiomer. The higher binding constant of the R-ketoprofen-β-CD complex can be explained by the reduction in angle between the two rings and the lower mobility of the carboxylic group that favored the formation of hydrogen bonds with β-CD. Nevertheless, the hydrophobic interactions were identified as the main factor and electrostatic interactions as the minor ones, contributing to ketoprofen’s enantioselectivity by utilizing β-CD.

The significant differences in calculated biding constants between R- and S-ketoprofen support the Raman findings and evidence the ability of β-CD as a chiral selector for R- and S-ketoprofen.

However, these findings have not been supported by the outcome of the previous study where the application of β-CD to discriminate enantiomers of ketoprofen using circular dichroism (CD), isothermal titration calorimetry (ITC), and NMR analysis has been explored [84]. Based on the results, only minor structural changes in complexes between enantiomers have been observed, leading to the conclusion that the ability of β-CD to serve as a chiral selector in this case is limited. Furthermore, K values for both enantiomers have been calculated to be 791 ± 19 M^−1^ and 568 ± 8 M^−1^ for S-, and R-enantiomer respectively, using ITC measurements. It is worth mentioning that due to low KP concentration and binding affinity, the critical parameter *c*, which determines the shape of the binding isotherm, has been below the optimal range. Thus, for differences in K values less than 2-fold, no precise distinction can be made, and hence, no chiral discrimination can be proved. However, the NMR and CD analyses have remarked small structural differences in diastereomeric complexes between enantiomers. The application of the molecular modeling approach has allowed to assess the structure of (R/S)-KP-β-CD complexes, following the experimental results. The stability constants cannot be compared between studies due to the application of a 10 times lower concentration of KP compared to the previous study [65], resulting in higher uncertainty results in ITC analysis.

#### 7.7.2. Ephedrine Derivatives

In the paper [50], the authors investigated the influence of binding constant and electrophoretic mobilities of complexes of four β-CD derivatives with phenethylamine enantiomers on the migration order and enantioselectivity. Although nonlinear and linear approaches to the binding constant determination have been tested, nonlinear regression was used to calculate the binding constant and further analysis since it was found to be more accurate and precise. Based on the research, the calculated electrophoretic complex mobility values have maintained the migration order, whereas for phenethylamine enantiomers, the binding constants have not been directly correlated with enantioselectivity in capillary electrophoresis. In some cases, enantiomers with higher affinity to cyclodextrin migrated faster. For instance, better enantioseparation of enantiomers of pseudoephedrine was observed using (2-hydroxypropyl)-β-CD (HP-β-CD) than β-CD even though β-CD was characterized by the higher value of binding constant, and obtained differences in K value between both enantiomers for HP-β-CD and β-CD were 2.5 and 33.7, respectively.

Nevertheless, it is worth noticing that the difference between the binding constant of two enantiomers seems to be the more predicting factor than the absolute values of the binding constant. In this study, the highest enantioseparation has been observed for pseudoephedrine with all tested CDs, including native β-CDs. In contrast, the ephedrine and methylephedrine showed only poor enantioseparation with all four investigated cyclodextrins. Focusing on complexes with β-CDs, pseudoephedrine enantiomers were characterized by the highest ΔK and higher electrophoretic selectivity compared to ephedrine, methylephedrine, and norephedrine. Since the apparent binding constants obtained through affinity capillary electrophoresis (ACE) can differ from the thermodynamic binding constant, for ephedrine enantiomers, isothermal titration calorimetry (ITC) measurements have been carried out. The results from ITC were in good agreement with data obtained by ACE, suggesting no or minimal impact of the capillary surface adsorption on the accuracy of determination of binding constant, indicating the reliability and consistency of the determination of binding constant values across those two methods. The thermodynamic results revealed that the formation of ephedrine-β-CD complex is an enthalpy-driven process, and the complexation of (-)-ephedrine with β-CD is more exothermic compared to (+)-ephedrine.

According to the different studies of chiral discrimination of ephedrine and N-methylephedrine enantiomers [47], it has been exposed that the application of native β-CD was capable of discrimination between enantiomers based on significant differences in chemical shifts in the NMR of single enantiomers. Furthermore, the Nuclear Overhauser Effect (NOE) study on inter- and intramolecular interaction supports findings that the complexes of (+)- and (-)-enantiomers with β-CD differed in host–guest interactions. However, no conformational changes have been observed upon complexation, and the calculated dissociation constants for N-methylephedrine complexes have been determined to be slightly different, with the uncertainties overlapping. Therefore, it is probable that the chiral selectivity is caused by the distinct orientation of the guest within the cavity, rather than the differences in conformation and complex stability. These findings may suggest that other factors beyond variation of complex stability may contribute to the chiral discrimination, and the prediction of migration order in CE analysis is challenging based on ROESY experiments.

Based on ITC analysis for ephedrine and pseudoephedrine at a pH value of 6.9 [85], the order of stability has been well reproduced, and the α values have been very similar to the results reported in the study [50], even though the latter study has been performed at the pH = 3. However, both tested compounds are basis, and applied pH values were below pKa.

#### 7.7.3. Thalidomide

In the next study [76], cyclodextrin-bonded stationary phases in reversed-phase high-performance liquid chromatography have been used to enable the enantioselective separation of thalidomide (THA) enantiomers. Among different tested CDs, native β-CD was identified as the most effective chiral selector. In order to evaluate the optimal conditions for enantioseparation, orthogonal experimental designs were applied. It has been noted that among the investigated three variables, the concentration of the organic modifier, namely acetonitrile, mainly impacts the chiral selection of THA enantiomers. The other factors, such as temperature and flow rate, play also important roles in this process, affecting the retention times. Since neither THA nor β-CD have ionizable groups at pH from 3 to 7, the pH variation in that range had no impact on the effectiveness of enantioseparation of THA. To further investigate the temperature dependence on retention and selectivity of THA enantiomers, the thermodynamic parameters have been calculated. Based on findings, the process is enthalpy-driven, and with the increase of temperature in the tested range (278–308 K), a decrease in retention time and effectiveness of enantioseparation has been observed. Additionally, NMR spectroscopy and quantum chemical molecular modeling studies were performed to investigate the conformation of THA and the type of interactions between THA enantiomers and β-CD. The results of complexation energy revealed that S-enantiomer bound stronger to β-CD than R-THA, which was in accordance with chiral liquid chromatography analysis. The results have shown that differences in affinity have a crucial role in the enantioseparation of THA enantiomers, and they have emphasized the ability of molecular modeling methods to predict the complexation and elution order of enantiomers. The enantioselectivity of THA has been also tested with the application of β-CD in the mobile phase in HPLC [81]. The obtained results have been accorded to the study that β-CD constitutes a chiral stationary phase [76], and the obtained binding constants for both studies were in the same order of magnitude.

#### 7.7.4. Propranolol

In the case of propranolol [73], it has been noted that the addition of a third component can significantly improve resolution in capillary zone electrophoresis and improve chiral selection by forming ternary complexes with β-CD and propranolol. However, it can simultaneously lead to a decrease in values of binding constants calculated from fluorescence studies and retention time. The improvement in chiral recognition with commodifiers can be attributed to the greater reduction of K value for S-enantiomer complexes than for R-enantiomer, resulting in differences. However, the obtained K values for ternary complexes are too small and have a high degree of error, making them impractical for further analysis.

In another study that investigated propranolol, room temperature phosphorescence (RTP) has been applied [67]. The presence of other components allowed the more stable complexes with β-CD to be formed with bromocyclohexane addition, serving as a heavy atom and increasing the intensity of the RTP. As a result, the β-CD can be applied as a chiral selector to distinguish enantiomers of propranolol in the rapid and simple method which is RTP. The authors of the next work concluded that based on ITC measurement at pH = 4.8, no chiral recognition has been observed for propranolol without the addition of a third component [83].

#### 7.7.5. Naproxen

In some cases, enantiomers do not differ much, sometimes only by the disposition of one group like in the case of naproxen in which R- and S- isomers differ only by the position of a small methyl group [66]. Hence, chiral discrimination of these compounds can be a challenging task and requires extra adjustment of conditions of chiral analysis [29,55,75] or even incorporation of other components [67,73,78] to improve the enantioselectivity of β-CDs. For naproxen (Nap), the inclusion of 1-iodobutane (Ibu) to form ternary complexes with β-CDs resulted in significant improvement of enantiodiscrimination, by application of room temperature phosphorescence (RTP) analysis [66]. The calculated values of formation constant between binary Nap/β-CD and ternary Nap/β-CD/Ibu complexes differ significantly. The values for binary complexes are (8.97 ± 0.30) × 10^2^ and (8.21 ± 0.25) × 10^2^ M^−1^, whereas for ternary (8.02 ± 0.15) × 10^3^ and (2.50 ± 0.06) × 10^3^ M^−1^ for (R)-Nap and (S)-Nap, respectively. It is worth mentioning that the α ratio increased almost 3 times with the presence of a third component, and ternary complexes exhibited enantiomeric differentiation in both RTP intensity and RTP lifetime. The Ibu incorporation, serving as a hydrophobic space-regulator, can enhance the fit of Nap evolving stronger interactions, and since R- and S-Nap fit differently into the cavity of β-CD, improving chiral discrimination of naproxen enantiomers.

The authors of the next work also applied RTP analysis to assess the binding constant of Naproxen enantiomers [68]. Accordingly with the previous work [66], since the α ratio for binary complexes was only 1.13, little enantioseparation was observed without a third component. The absolute differences values reported in both studies were in the same order of magnitude; however, association values were different, 897 ± 30, 821 ± 25 M^−1^ [66] and 502, 566 M^−1^ [68], for R- and S-enantiomer, respectively. The discrepancy between both results may exist due to the difference in excitation wavelength used for fluorescence lifetime measurements. It was noticed that the excitation energy can impact phosphorescence decay, therefore obtaining results from fluorescence measurements [114]. Since both studies indicate reverse order in the stability of the complexes of naproxen in the presence and without a third component, further examination is required to precisely assess the β-CD affinity towards each enantiomer. The results of molecular modeling calculations performed in the study [68] have indicated that S-enantiomer creates stronger interactions with β-CD with energy differences around 4.2 kJ/mol.

#### 7.7.6. Noradrenaline

The authors of the next work have applied NMR analysis to study chiral recognition of enantiomers of noradrenaline [36]. Regarding obtained K values, it has been concluded that no enantioselectivity has been observed. These observations have been in line with the study using CE methods [50,115,116]. Although no chiral recognition has been observed, the obtained binding constant between NMR and one of CE analyses differ significantly, namely Ks = 34.9 M^−1^ and K_R_ = 34.9 M^−1^ [50] and Ks = 537 M^−1^ and K_R_ = 516 M^−1^ [36] for CE and NMR studies, respectively. The variation in obtained values can be attributed to their underlying principles, sensitivity, applied solvent, and the analysis condition since NMR and CE have been performed at pH 7.0 [36] and 3.0 [50], respectively.

#### 7.7.7. Amino Acids

Amino acids are one of the smallest chiral molecules, and they can exist in various dissociated and protonated forms depending on the pKa values and pH of the solution. The studies of the complexation of amino acids to cyclodextrin have shown a wide range spectrum of obtained binding constants values, depending on applied pH value (see Table 6) and analytical methods. In [93], a series of amino acids were used to create complexes with native and modified β-CDs, and to test the effectiveness of these host molecules as chiral selectors. Based on the results from the potentiometric analysis, the values for L- and D-amino acids have been similar, within the range of measurement uncertainty. This led to the conclusion that no enantioseparation has been observed by using native β-CD regardless of applied analytical methods. The complexes formed by the fully protonated forms exhibit very low stability constants, whereas the more stable complexes have been created for anionic forms of aromatic amino acids. Remarkably, the neutral forms tended to form complexes of low K values, which is untypical compared to other organic acids containing aromatic groups. However, neutral amino acids exist not as chargeless molecules but rather as zwitterions that promote stronger hydration by interactions between water and guest molecules, leading to the formation of less stable complexes with β-CD.

It was noticed that the binding constant values for dipeptides are higher than for single amino acids, and in some cases, enantioseparation can be achieved [83], suggesting that amino acids are too small molecules to create strong interactions inside the cavity of β-CD. This finding can be supported by the fact that α-CD has been observed to be a more effective chiral selector for amino acids than β-CD [117].

The interaction between the enantiomers of dipeptides and β-CD, in their protonated and zwitterionic forms, has been investigated using CE and calorimetry at the temperature of 298.15 K [59]. Generally, binding constants for protonated forms were 1.5 times higher than for neutral species, and DD-enantiomers formed stronger complexes compared to LL-enantiomers. For, zwitterions, no significant differences have been observed. In CE analysis conducted at low pH, less stable complex, i.e., with LL-enantiomer, migrated faster. However, due to the almost identical stability of the complexes for both enantiomers in their neutral forms, the migration order was determined by the mobility of the complexes, resulting in a reversal in migration order at pH 3.8. The calculated K_+_ values (for the protonated forms) for alanylphenylalanine (Ala-Phe) were 42 ± 5 and 34 ± 4 M^−1^, for D-Ala-D-Phe and L-Ala-L-Phe, respectively. Those values were in agreement with estimated apparent pH-independent K_+_ values of 44 and 42 M^−1^ for DD- and LL-enantiomer, respectively [60]. The K _±_ values (for zwitterionic forms) have shown variation across different studies. In [59], the K _±_ values were placed in the range of 18–27 M^−1^, while in [60], the pH-independent K values ranged from 2–4 M^−1^ based on CE analysis.

The structure of analyzed amino acids can significantly influence the affinity towards β-CD. Based on potentiometric measurements, the aliphatic amino acids such as alanine, valine, and norvaline were unable to form stable complexes with β-CD due to their low hydrophobicity [94]. Therefore, the K values were too small to be measured. Contrarily, the aromatic amino acids were more likely to form complexes with β-CD, e.g., tryptophan (Trp). The order of stability of complexes with Trp enantiomers has been in line with the previously mentioned outcomes of fluorescence [69] and ultrafiltration methods [96]. Beyond the high level of uncertainty of K values obtained by the application of the potentiometric method, the significant differences between enantiomers allowed to assess the enantioselectivity of β-CD toward the tryptophan enantiomers [94].

The application of native β-CD as a chiral selector has been tested in the case of larger molecules, i.e., amino acids containing additional groups, e.g., protecting groups such as benzyloxycarbonyl substituent (Z or Cbz) [86,87]. Z-protected amino acids are widely used in peptide synthesis. Based on NMR analysis, it has been noticed that the phenyl group of the Z-part of the molecules was inserted into the β-CD cavity since it is the most hydrophobic part of the tested compounds [86]. Z-Dipeptide of Z-protected glutamic acid and tyrosine (Z-Glu-Tyr) was characterized by higher affinity to β-CD than single Z-Glu leading to modest enantioselectivity with a ratio of 1.0–1.3 and 0.7–1.1 for dipeptide and Z-Glu, respectively. Regarding Z-Glu molecules, although variations in ΔH and ΔS have been noticed between enantiomers, the disparities in ΔG were not substantial due to enthalpy–entropy compensation [87]. While N-acetyl-tyrosine has shown greater negative values of ΔG compared to the studied dipeptide [83], no chiral recognition has been found, as indicated by a ratio within the range of 0.8–1.1 [86]. Nevertheless, for the Z-dipeptide, γ-CD demonstrated higher effectiveness with a ratio of 2.43 compared to β-CD, which can be attributed to the better fit of the cavity size to the guest molecules.

The chiral analyses for dansylated and DNP (Dinitrophenyl)-amino acids have been also performed. For dansylated-glutamate (Dns-Glu), the CE analysis has indicated that the average α ratio was approximately equal to unity [62]. Whereas the suggested structures of DNP-L-amino acids and DNP-D-amino acids provide detailed information about the specific chiral discrimination interaction and the chirality forces that are responsible for chiral resolutions [45]. It has been observed that the DNP group is essential for chiral recognition since it formed a stable inclusion complex with the β-CD cavity. Moreover, the alkyl groups of amino acids contribute significantly to chiral recognition by either forming a secondary inclusion complex with another β-CD cavity (in the case of DNP-L-amino acids) or causing steric repulsion with the hydroxyl groups at the edge of the cavity (in the case of DNP-D-amino acids). Therefore, the formation of a secondary inclusion complex between β-CD and the alkyl groups of DNP-D-amino acids was not feasible. Regardless of the analytical methods used to calculate the dissociation constants, the results consistently demonstrate the ability of β-CD to form enantioselective complexes with tested DNP-amino acids (Table 7).

According to the mentioned examples, the effectiveness of β-CD as a chiral selector for amino acids depends strongly on the size and chemical properties of guest molecules as a consequence applied conditions of analysis especially the pH of the solutions.

## 8. Conclusions

As shown in this review, native β-CD is frequently used as a chiral selector in various analytical methods due to its unique ability to form stereoselective inclusion complexes with various compounds. Moreover, β-CD can act as a chiral selector both as a part of chiral stationary phase or as a component of a solution.

It has been shown that the chosen analytical method and applied conditions, i.e., temperature, pH, concentration of chiral selectors, buffer, applied solvent or the presence of modifiers can impact the results or even control whether chiral recognition occurs. Furthermore, the effectiveness of native β-CD may depend on the size and the physicochemical properties of the tested molecules. It has been remarked that the molecules that match the size of the cavity and fit more rigid tend to form more stable complexes with β-CD due to multiple binding sites of a cavity that guests can interact with and the improvement in the strength of interactions. Therefore, the addition of a third component can contribute to the occurrence of chiral selectivity by creating more rigid structures of complexes.

Although the effectiveness of chiral discrimination using β-CD depends on numerous factors, as stated above, several descriptors of guest structure can ease the assessment of whether chiral recognition is likely to occur. The compound with more rigid and bulky groups tends to display better chiral recognition than more flexible molecules that can adjust more freely to the cavity of β-CD. The presence of a hydrophobic group inside the cavity of the host promotes the formation of stereoselective host–guest interactions. The guests that better fit the cavity size tend to exhibit higher enantioselectivity due to rigid movement inside the cavity. The main limitations of using native CDs as chiral selector are related to their relatively low solubility in comparison to substituted CDs. Additionally, the structural homogeneity of native CDs restricts their ability of creation various types of interactions with guests and their application as chiral selector is mostly limited hydrophobic or moderately hydrophilic chiral molecules. However, native CDs are characterized by lower production costs, they are biodegradable and under optimized conditions of analysis; they can be successfully used as chiral selectors in various analytical methods.

Unfavorably, in some cases, the relatively high uncertainties to the mean values of calculated binding constants preclude unequivocally determination of the differences between the stability of the formed diastereomeric complexes between enantiomers and β-CD. Therefore, the assessment of whether β-CD interacts differently with one of the isomers and can be successfully used as a chiral selector is impossible. To enhance the accuracy of the binding constant determination, it is crucial to thoughtfully select experimental conditions. One of the more important factors that can influence the attained resolution, thus the effectiveness of the enantioselectivity and the accuracy of determination binding constants, can be the concentration of the chosen chiral selector. If the concentration is insufficient, the chiral recognition may not occur due to the limited β-CD molecules accessible to selectively interact with chiral molecules, even though the differences in binding constant between isomers can be significant. However, the excessive concentration of native β-CD can, among other things, impact the solubility and viscosity, increase the separation time of the analysis or promote the formation of undesired 1:n complexes leading to unsatisfactory outcomes. Beyond the huge advancement in this field, since the differences between binding constants for enantiomers can be relatively subtle, the more enantioselective and more precise methods of analysis are still required to achieve satisfactory discrimination of enantiomers including the range of uncertainty of the measurements.

Concluding, the question “Can the enantiomers of the selected compounds be effectively separated using β-CD?” is more complex than it appears. In most cases, the answer is “yes, but only under specific conditions”. Understanding the relationship between the physicochemical properties of solute, the type of interaction involved in the formation of stable complexes with β-CD and the desired conditions of analysis can provide insights into the expected values and can aid in evaluating the optimal conditions of the analysis.

## Figures and Tables

**Table 1:** The selected methods of the determination of binding constant applied in the reviewed papers in descending order of number of works.

Analytical Method	Principle	Time of Analysis	Complexity	Sensitivity	Destruction of the Sample	Spectrum of Compounds	Other Aspects, Requirements	Number of Works	Ref.
NMR Spectroscopy	Change in chemical shift upon binding	Moderate to long	High	Moderate	No	Very broad	Expensive deuterated solvents, maintenance of the spectrometer, Provide detailed information on the structure	19	[29,30,31,32,33,34,35,36,37,38,39,40,41,42,43,44,45,46,47]
Capillary Electrophoresis (CE)	Differences in electrophoretic mobility and interaction with capillary	Fast	Moderate	Very high	No	Polar compounds	Allows separation, Requires optimization of conditions	16	[48,49,50,51,52,53,54,55,56,57,58,59,60,61,62,63,64]
Fluorescence Spectroscopy (FS) and anisotropy	Emission of the light after excitation	Fast to moderate	Low to moderate	High	No	Fluorescent compounds	May require additional compounds	10	[65,66,67,68,69,70,71,72,73,74]
High-Performance Liquid Chromatography (HPLC) and Liquid Chromatography (LC)	Interaction with the stationary or/and mobile phases	Fast to moderate	Moderate to high	High	Yes	Very broad	Requires optimization of conditions, May require a column with β-CD bounded allows separation	9	[49,75,76,77,78,79,80,81,82]
Isothermal Titration Calorimetry (ITC)	Heat released/absorbed upon complexation	Fast	Moderate	High	No	Very broad	Allows direct determination of thermodynamic binding constant and ΔH, ΔS,	8	[50,59,83,84,85,86,87,88]
UV–vis Spectroscopy	Absorption of light in the UV–vis spectra	Fast	Low	Moderate	No	Broad, chromophores	Limited information on molecular interactions	6	[44,45,65,89,90,91]
Circular Dichroism (CD) Spectroscopy	Absorption of circularly polarized light	Fast to moderate	moderate	Moderate to low	No	Broad, optical active	Limited to chiral molecules	5	[44,45,74,84,92]
Potentiometry	Change in electrochemical potential	Fast to moderate	Low to moderate	Moderate	No	Ionic compounds	Requires calibration and standardization	2	[93,94]
Gas Chromatography (GC)	Interaction with the stationary phase, volatility	Fast to moderate	Moderate to high	High	Yes	Volatile compounds	Allows separation, Requires optimization of conditions	1	[95]
Ultrafiltration	Separation of molecules depending on the size	Fast to moderate	Low to moderate	Moderate	No	Large molecules	Simple method, Membrane selectivity	1	[96]

**Table 2:** Selected articles containing binding constants determined for each pair of enantiomers using native β-CD as a chiral selector.

No.	Guest Molecule	Enantiomers	K [M^−1^]	ΔK [M^−1^] Binding Selectivity (α) *	Analytical Methods Used for the Determination of Binding Constant	Conditions (pH and Temp) at Which the Binding Constants Were Determined	STOICHIOMETRY of the Complex	Chiral Stationary Phase/β-CD Added to the Solution	Other Analytical Approaches, Molecular Modeling	Year	Ref.
1.	Ketoprofen	(R)-(-)-ketoprofen (S)-(+)-ketoprofen	(R): 2750 (UV) (S): 1299 (UV) (R): 4088 (FLUO) (S): 2547 (FLUO)	ΔK = 1451 (UV) α = 2.12 (UV) ΔK = 1541(FLUO) α = 1.61 (FLUO)	UV, Fluorescence (Benesi–Hildebrand (BH) equation)	303.15 K pH = 7	1:1	Added to the solution	Raman, FTIR, TD	2020	[65]
2.	Ephedrine	(1R,2S)-(-)-ephedrine (1S,2R)-(+)-ephedrine	(-): 35.7 (CE) (+): 34.2 (CE) (-): 37.4 (ITC) (+): 33.5 (ITC)	ΔK = 1.5 (CE) α = 1.04 (CE) ΔK = 3.9 (ITC) α = 1.12 (ITC)	CE (nonlinear regression) ^a^ ITC	298.15 K pH = 3	1:1	Added to the solution	TD	2017	[50]
3.	Pseudoephedrine	(1S,2S)-(+)-pseudoephedrine (1R,2R)-(-)-pseudoephedrine	(+): 127.2 (-): 93.5	ΔK = 33.7 α = 1.36	CE	298.15 K pH = 3	1:1	Added to the solution		2017	[50]
4.	Methylephedrine	(1S,2R)- (+)-methylephedrine (1R,2S)-(-)-methylephedrine	(+): 96.8 (-): 94.6	ΔK = 2.2 α = 1.02	CE	298.15 K pH = 3	1:1	Added to the solution		2017	[50]
5.	Norephedrine	(1S,2R)- (+)-norephedrine (1R,2S)-(-)-norephedrine,	(+): 34.9 (-): 34.9	ΔK = 0 α = 1.00	CE	298.15 K pH = 3	1:1	Added to the solution		2017	[50]
6.	Tröger’s base (TB)	(-)Tröger’s base (+)Tröger’s base	(-): 303 ± 8 (+): 219 ± 8	ΔK = 84 ± 11 α = 1.38 ± 0.06	CE	298.15 K pH = 2.5	1:1	Added to the solution	NMR, X-ray	2016	[51]
7.	Thalidomide (THA)	(R)-(+)-thalidomide (S)-(-)-thalidomide	(S): 91 (R): 83	ΔK = 8 α = 1.10	HPLC	293.15 K pH = 5	1:1	Stationary	NMR titration, TD, molecular modeling	2016	[76]
8.	9-[2-(phosphonomethoxy)propyl]adenine (PMPA)	(R)-PMPA (S)-PMPA	(R): 46.4 ± 4.8 (S): 41.5 ± 7.8	ΔK = 4.9 ± 9.2 α = 1.12 ± 0.24	CE (nonlinear regression) ^a^	298.15 K pH = 10	1:1	Added to the solution		2016	[52]
9.	9-[3-hydroxy-2-(phosphonomethoxy)propyl]adenine (HPMPA)	(R)-HPMPA (S)-HPMPA	(S): 27.0 ± 8.6 (R): 25.6 ± 8.5	ΔK = 1.4 ± 12.1 α = 1.06 ± 0.50	CE (nonlinear regression) ^a^	298.15 K pH = 10	1:1	Added to the solution		2016	[52]
10.	9-[2-(phosphonomethoxy)propyl]-2,6-diaminopurine (PMPDAP)	(R)-PMPDAP (S)-PMPDAP	(R): 21.7 ± 4.6 (S): 19.5 ± 4.1	ΔK = 2.2 ± 6.1 α = 1.11 ± 0.33	CE (nonlinear regression) ^a^	298.15 K pH = 10	1:1	Added to the solution		2016	[52]
11.	(1-(1-(2,6-diamino-9H-purin-9-yl)-3-hydroxypropan-2-yl)-1,2,3-triazol-4-yl)phosphonic acid (ANP 4)	(R)-ANP 4 (S)-ANP 4	(S): 13.7 ± 5.6 (R): 13.3 ± 3.3	ΔK = 0.4 ± 6.5 α = 1.03 ± 0.49	CE (nonlinear regression) ^a^	298.15 K pH = 10	1:1	Added to the solution		2016	[52]
12.	2-((diisopropoxyphosphoryl)methoxy)-3-(2,4-dioxo-3,4-dihydropyrimidin-1(2H)-yl)propanoic acid (ANP 5)	(R)-ANP 5 (S)-ANP 5	(S): 37.8 ± 5.7 (R): 35.5 ± 5.6	ΔK = 2.3 ± 8.0 α = 1.07 ± 0.23	CE (nonlinear regression) ^a^	298.15 K pH = 10	1:1	Added to the solution		2016	[52]
13.	α-cyclohexylmandelic acid (CHMA)	(-)-CHMA (+)-CHMA	(-): 30.52 (+): 17.47	ΔK = 13.05 α = 1.75	ICD	283.15 K pH = 3.51	1:1	Added to the solution	HSCCC, TD	2015	[92]
14.	α-terpineol	(+)-α-terpineol (−)-α-terpineol	(+): 413 ± 10 (-): 399 ± 8	ΔK = 14 ± 13 α = 1.04 ± 0.03	HPLC	298.15 K pH = 7	1:1	Added to the solution	TG, X-ray, molecular modeling	2015	[77]
15.	Paliperidone Propranolol Risperidone Verapamil	R-isomer S-isomer	Details can be found in Section 7.5.	Details can be found in Section 7.5.	CE (linear model, y- reciprocal equation)	298 K pH = 2.5 and 7.0	1:1	Added to the solution	NMR (only for risperidone)	2013	[53]
16.	Cyclopentolate (CP)	(R)-cyclopentolate (S)-cyclopentolate	204.2/138.6	ΔK = 65.6 α = 1.47	HPLC	293.15 K pH = 2.5	1:1	Added to the solution		2013	[78]
17.	2,6-diketopiperazine derivative (2,6-DKP)	(R,R)-2.6-DKP (S,S)-2,6-DKP	19.5/12.4	ΔK = 7.1 α = 1.57	HPLC	293.15 K pH = 2.5	1:1	Added to the solution		2013	[78]
18.	Methylphenobarbital (MPB)	(R)-MPB (S)-MPB	171.3/149.7	ΔK = 21.6 α = 1.14	HPLC	293.15 K pH = 2.5	1:1	Added to the solution		2013	[78]
19.	2-phenyl-2,3-dihydro- 4H-chromen-4-onen (FL)	(+)-flavanone (-)-flavanone	222/184	ΔK = 38 α = 1.21	NMR	298.15 K ^b^ pH = 7	1:1	Added to the solution	TD	2013	[31]
20.	(2-(2-hydroxyphenyl)-2,3-dihydro-4H-chromen-4-one (2′OHFL)	(+)-2′-hydroxyflavanone (-)-2′-hydroxyflavanone	NA ^c^	NA ^c^	NMR	298.15 K ^b^ pH = 7	1:1	Added to the solution		2013	[31]
21.	Naproxen	(R)-(-)-naproxen (S)-(+)-naproxen	(R): 897 ± 30 (S): 821 ± 25 (R) ^d^: (8.02 ± 0.15) × 10^3^ (S) ^d^: (2.50 ± 0.06) × 10^3^	ΔK = 76 ± 39 α = 1.09 ± 0.05 ΔK ^d^ = (5.52 ± 0.16) × 10^3^ A ^d^ = 3.21 ± 0.10	RTP	298.15 K pH = 2.78	1:1 1:1:1 ^d^	Added to the solution	UV	2013	[66]
22.	Propranolol	(R)-(+)-propranolol (S)-(-)-propranolol	(R): 924.84 (S): 877.94 (R) ^e^: 6.44 × 10^3^ M^−3^ (S) ^e^: 4.56 × 10^3^ M^−3^	ΔK = 46.9 α = 1.05 ΔK ^e^ = 1.88 × 10^3^ α = 1.41	RTP	298.15 K pH = 3.0	NP	Added to the solution	UV–vis, NMR,	2013	[67]
23.	Series of amino acids: Phenylalanine (Phe) Tyrosine (Tyr) Tryptophan (Trp) Leucine (Leu) Asparagine (Asp) Glutamic acid (Glu) Histidine (His) Threonine (Thr)	L-amino acid D-amino acid	Details can be found in Section 7.7.7.	Details can be found in Section 7.7.7.	Potentiometry	298.15 K pH range varied for each amino acid	1:1	Added to the solution	UV–vis, NMR	2012	[93]
24.	2-(4-hydroxyphenyl)-2,3-dihydro-4H-chromen-4-one (4′OHFL)	(+)-4′-hydroxyflavanone (-)-4′-hydroxyflavanone	254/145	ΔK = 109 α = 1.75	NMR	298.15 K ^b^ pH = 7	1:1	Added to the solution	TD	2012	[32]
25.	Sibutramine (SIB)	(R)-sibutramine (S)-sibutramine	(R): 228 ± 4 (S): 199 ± 4	ΔK= 29 ± 5.7 α= 1.14 ± 0.03	CE (linear model, x-reciprocal equation)	298.15 K pH = 4.3	1:1	Added to the solution		2011	[48]
26.	Naproxen	(R)-(-)-naproxen (S)-(+)-naproxen	(R): 502 (S): 566 (R) ^f^: 2.43 × 103 M^−1^ (S) ^f^: 3.20 × 103 M^−2^	ΔK = 64 α = 1.13 ΔK = NA α = NA	RTP	298.15 K pH = 3.0	1:1 1:1:1 ^f^ (R-enantiomer) 1:1:2 ^f^ (S-enantiomer)	Added to the solution	NMR, molecular modeling	2011	[68]
27.	Ketoprofen	(R)-(-)-ketoprofen (S)-(+)-ketoprofen	(S): 1098.54 ± 175.36 (CD) (R): 758.58 ± 69.89 (CD) (S): 791 ± 19 (ITC) (R): 568 ± 8 (ITC)	ΔK = 339.96 ± 188.77 (CD) α = 1.45 ± 0.27 (CD) ΔK = 223 (ITC) α = 1.39 ± 0.04 (ITC)	CD ITC	298.15 K pH = 7.4	1:1	Added to the solution	NMR, ROESY, molecular modeling, photolysis, ESI-MS	2011	[84]
28.	Ibuprofen	(R)-(-)-ibuprofen (S)-(+)-ibuprofen	(R): 2100 (S): 2040	ΔK = 60 α = 1.03	UV (linear regression)	298.15 K pH = NP	1:1	Added to the solution	NMR	2011	[89]
29.	Ala-Phe	D-Ala-D-Phe L-Ala-L-Phe	DD_(+)_^l^:80 ± 13 LL_(+)_^l^: 48 ± 8 DD_(±)_^l^:24 ± 3 LL_(±)_^l^:19 ± 2	ΔK_(+)_^l^ = 32 ± 15.2 α_(+)_^l^ = 1.67 ± 0.39 ΔK_(±)_^l^ = 5 ± 3.6 α_(±)_^l^ = 1.26 ± 0.21	ITC	298.15 K pH = 2.2 and 3.8	1:1	Added to the solution	CE, TD	2010	[59]
30.	Ala-Tyr	D-Ala-D-Tyr L-Ala-L-Tyr	DD_(+)_^l^:76 ± 9 LL_(+)_^l^:48 ± 8 DD_(±)_^l^:52 ± 9 LL_(±)_^l^:39 ± 7	ΔK_(+)_^l^ = 28 ± 12.0 α_(+)_^l^ = 1.58 ± 0.32 ΔK_(±)_^l^ = 13 ± 11.1 α_(±)_^l^ = 1.33 ± 0.33	ITC	298.15 K pH = 2.2 and 3.8	1:1	Added to the solution	CE, TD	2010	[59]
31.	Permethrin	1R-cis-permethrin 1S-cis-permethrin	179.14 ^g^/160.70	ΔK = 18.44 α = 1.12	HPLC	298 K pH = NP	NP	Stationary	TD	2010	[79]
32.	Permethrin	1R-trans-permethrin 1S-trans-permethrin	92.18 ^g^/79.65	ΔK = 12.53 α = 1.16	HPLC	298 K pH = NP	NP	Stationary	TD	2010	[79]
33.	Brompheniramine	(+)-brompheniramine (-)-brompheniramine	(+): 192.2 ^k^ (HPLC) (-): 183 ± 9 (HPLC) (+): 146.6 ^k^ (CE) (-): 137 ± 0.5 (CE)	ΔK = 9.2 (HPLC) α = 1.05 (HPLC) ΔK = 9.6 (CE) α = 1.07 (CE)	HPLC CE	298 K pH = 7 (HPLC) and pH = 2.5 (CE)	1:1	Added to the solution		2009	[49]
34.	Cyclopentolate	(+)-cyclopentolate (-)-cyclopentolate	268.7 ^k^/202 ± 7 (HPLC) 1367.5 ^k^/ 1036 ± 22 (CE)	ΔK = 66.7 (HPLC) α = 1.33 (HPLC) ΔK = 331.5 (CE) α = 1.32 (CE)	HPLC CE	296–298 K pH = 7 (HPLC) and pH = 2.5 (CE)	1:1	Added to the solution		2009	[49]
35.	Tryptophan (Trp)	D-tryptophan L-tryptophan	L: 297.3 D: 112.9	ΔK = 184.4 α = 2.63	FS	298.2 K pH = 10.4	1:1	Added to the solution	ESI-MS	2009	[69]
36.	1,1′-binaphthol (BINOL)	(R)-1,1′-binaphthol (S)-1,1′-binaphthol	R: 404 ± 98 (298.15 K) S: 352 ± 49 (298.15 K)	ΔK = 52 ± 110 α = 1.15 ± 0.32	FS (non-linear) ^a^	278.15–318.15 K pH = NP	1:1	Added to the solution	TD, molecular modeling	2008	[70]
37.	Promethazine hydrochloride (PTZ)	(R)-promethazine (S)-promethazine	(2.4 ± 0.4) × 10^3^/(1.3 ± 0.1) × 10^3^	ΔK = (1.1 ± 0.4) × 10^3^ α = 1.85 ± 0.34	NMR	298 K pH = NP	1:1	Added to the solution	Fluorescence, UV, molecular modeling	2008	[33]
38.	2-acetyl-1-(4′-chlorophenyl)-6,7-dimethoxy-1,2,3,4-tetrahydroisoquinoline (AA)	(R)-AA (S)-AA	(R): 15,889 (S): -	ΔK = NA α = NA	UV–vis phase solubility diagram	298.15 K pH = NP	1:1	Added to the solution	FTIR-ATR, molecular modeling	2008	[90]
39.	1,1′-binaphthyl-2,2′-diyl hydrogen phosphate (BNP)	(S)-BNP (R)-BNP	(S): 369.6 (R): 230.2	ΔK = 139.4 α = 1.61	FS	T = 298 K pH = 6.9	1:1	Added to the solution	TD	2007	[71]
40.	Bifonazole	(R)-bifonazole (S)-bifonazole	2767 ± 552/2767 ± 552	ΔK = 0 ± 781 α = 1.00 + 0.28	CE (γ-reciprocal equation)	T = 288.15 pH = 3.0	1:1	Added to the solution		2007	[54]
41.	Miconazole	(R)-miconazole (S)-miconazole	932 ± 120/749 ± 91	ΔK = 183 ± 151 α = 1.24 ± 0.22	CE (γ-reciprocal equation)	T = 288.15 pH = 3.0	1:1	Added to the solution		2007	[54]
42.	Econazole	(R)-econazole (S)-econazole	737 ± 121/622 ± 97	ΔK = 115 ± 155 α = 1.19 ± 0.27	CE (γ-reciprocal equation)	T = 288.15 pH = 3.0	1:1	Added to the solution		2007	[54]
43.	Sulconazole	(R)-sulconazole (S)-sulconazole	2394 ± 640/2357 ± 611	ΔK = 37 ± 886 α = 1.02 ± 0.38	CE (γ-reciprocal equation)	288.15 K pH = 3.0	1:1	Added to the solution		2007	[54]
44.	Tryptophan (Trp)	L-Trp D-Trp	L: 4.3 × 10^−5^ D: 3.1 × 10^−5^	ΔK = 1.2 × 10^−5^ α = 1.39	ultrafiltration	298.15 K pH = 6.0	NP	Stationary	UV–vis	2007	[96]
45.	Citalopram (CT)	(R)-citalopram (S)-citalopram	R: 418 S: NP	ΔK = NA α = NA	NMR	298.15 K pH = 7.0	1:1	Added to the solution	ROESY	2007	[34]
46.	6-[imidazol-1-yl(phenyl)methyl]-3-methyl-1,3-benzothiazol-2-one [IB]	(R)-(+)-IB (S)-(-)-IB	R: 215–242 ^h^ S: 154–185 ^h^	ΔK = 56–85 ^h^ α = 1.3–1.54 ^h^	NMR	298 K pH = 2.5	1:1	Added to the solution	ROESY, ESI-MS	2006	[35]
47.	Alprenolol Isoproterenol Isoxsuprine Metaproterenol Methoxamine Nadolol Pindolol Propranolol Ritodrine Terbutaline	(R)-isomer (S)-isomer	Details can be found in Section 7.4.	Details can be found in Section 7.4.	CE	T- 288.15–303.15 K pH = 2.5	1:1	Stationary		2006	[55]
48.	Isomenthone	(+)-isomenthone (−)-isomenthone	(+)_I_: 6.9 ^i^ (−)_I_: 7.4 ^i^ (+)_II_: 15 ^i^ (−)_II_: 13 ^i^	ΔK_I_ = −0.5 ^i^ α_I_ = 0.93 ΔK_II_ = 2 ^i^ α_II_ = 1.22 α_I_α_II_ = 1.14	GLC	T = 333 K pH = NP	1:1 1:2	Stationary Stationary+Added to the solution	TD	2005	[95]
49.	Fenchone	(-)-fenchone (+)-fenchone	(-)_I_: 9.9 ^i^ (+)_I_: 12 ^i^ (-)_II_: 57 ^i^ (+)_II_: 44 ^i^	ΔK_I_ = −2.1 ^i^ α_I_=0.85 ΔK_II_ = 13 ^i^ α_II_ = 1.29 α_I_ * α_II_ = 1.09	GLC	T = 333 K pH = NP	1:1 1:2	Stationary Stationary+ added to the solution	TD	2005	[95]
50.	6-[imidazol-1-yl(phenyl)methyl]-3-methyl-1,3-benzothiazol-2-one [IB]	(R)-(+)-IB (S)-(-)-IB	R: 322 S: 281	ΔK = 41 α = 1.15	CE	298 K pH = 2.5	1:1	Added to the solution	HPLC	2004	[56]
51.	Noradrenaline	(S)-(+)- noradrenaline (R)-(-)- noradrenaline	S: 537 R: 516	ΔK = 21 α = 1.04	NMR	T = 298.15 K pH = 7.0	1:1	Added to the solution	ROESY	2004	[36]
52.	Amino acids: Alanine, Valine, Norvaline, Histidine, Isoleucine	D-isomer L-isomer	D: - L: -	ΔK = NA α = NA	Potentiometry	298.15 K pH = 4–8 and pH > 11	1:1	Added to the solution		2004	[94]
53.	Ala-Phe	L-Ala-L-Phe D-Ala-D-Phe	LL_(-)_^l^: 403 ± 46 DD_(-)_^l^: 135 ± 51 LL_(±)_^l^: 274 ± 35 DD_(±)_^l^: 105 ± 45	ΔK_(+)_^l^ = 268 ± 68.7 α_(+)_^l^ = 2.98 ± 1.17 ΔK_(±)_^l^ = 169 ± 57.0 α_(±)_^l^ = 2.61 ± 1.17	Potentiometry	298.15 K pH = 4–8 and pH > 11	1:1	Added to the solution		2004	[94]
54.	Tryptophan (Trp)	L-Trp D-Trp	LL_(-)_^l^: 571 ± 102 DD_(-)_^l^: 140 ± 18 LL_(±)_^l^:447 ± 83 DD_(±)_^l^: 88 ± 17	ΔK_(+)_^l^ = 431 ± 103.6 α_(+)_^l^ = 4.08 ± 0.90 ΔK_(±)_^l^ = 359 ± 84.74 α_(±)_^l^ = 5.08 ± 1.36	Potentiometry	298.15 K pH = 4–8 and pH > 11	1:1	Added to the solution		2004	[94]
55.	Cinchonan-9-ol	Cinchonine ((9S)-Cinchonan-9-ol) Cinchonidine ((9R)-Cinchonan-9-ol)	S: 117 R: 108	ΔK = 9 α = 1.08	FS	T = 298.15 K pH = 7.2	1:1	Added to the solution	TD, NOESY	2003	[72]
56.	Melatonine	(+)-melatonin (-)-melatonin	(+): 91.86 (-): 79.68	ΔK = 12.18 α = 1.15	HPLC	NP	1:1	Added to the solution	TD	2003	[80]
57.	N-[2-(5-methoxy-1H-indol-3-yl)ethyl]cyclopropanecarboxamide	(+)-isomer (-)-isomer	(+): 115.50 (-): 100.42	ΔK = 15.08 α = 1.15	HPLC	NP	1:1	Added to the solution	TD	2003	[80]
58.	N-[2-(5-fluoro-1H-indol-3-yl)ethyl]acetamide	(+)-isomer (-)-isomer	(+): 158.77 (-): 143.37	ΔK = 15.4 α = 1.11	HPLC	NP	1:1	Added to the solution	TD	2003	[80]
59.	3-Hydroxy-2,2-dimethylcyclohexan-1-one	(-)-isomer (+)-isomer	(-): 350 ± 24 (+): 320 ± 20	ΔK = 30 ± 31 α = 1.09	NMR	303 ± 0.1 K pH = NP	1:1	Added to the solution	HR-DOSY, HPLC, ROESY, GC, GC-MS	2002	[30]
60.	3-Acetoxy-2,2-dimethylcyclohexan-1-one	(+)-isomer (-)-isomer	(+): 1010 ± 310 (-): 770 ± 100	ΔK = 240 ± 326 α = 1.31	NMR	303 ± 0.1 K pH = NP	1:1	Added to the solution	HR-DOSY, HPLC, ROESY, GC, GC-MS	2002	[30]
61.	3-Hydroxy-2-methyl-2-(2-propynyl) cyclohexan-1-one	(-)-isomer (+)-isomer	(-): 1225 ± 118 (+): 980 ± 90	ΔK = 245 α = 1.25	NMR	303 ± 0.1 K pH = NP	1:1	Added to the solution	HR-DOSY, HPLC, ROESY, GC, GC-MS	2002	[30]
62.	Z-Glu	L-Glu D-Glu	L: 86 ± 2 D: 84 ± 2	ΔK = 2 ± 2.83 α = 1.02 ± 0.03	ITC	298.15 K pH = 6.9	1:1	Added to the solution	TD	2001	[87]
63.	Dimethindene (DIM)	(R)-(-)-DIM (S)-(+)-DIM	R: 504 S: 457	ΔK = 47 α = 1.10	NMR	T = NP pH = 3.0	1:1	Added to the solution	ROESY, ESI-MS, CE, X-ray	2000	[37]
64.	Ethyl-phenylsulfoxide	(+)-ethyl-phenylsulfoxide (-)-ethyl-phenylsulfoxide	(+): 256.4 (-): 210.2	ΔK = 46.2 α = 1.22	NMR	303 ± 0.1 K pH = NP	1:1	Added to the solution	HPLC, HR-DOSY, ROESY,	2000	[38]
65.	N-acetyl- phenylalanine	N-acetyl-L-Phe N-acetyl-D-Phe	L: 67.5 ± 1.4 D: 60.7 ± 1.3	ΔK = 6.8 ± 1.9 α = 1.11 ± 0.03	ITC	298.15 K pH = 6.9	1:1	Added to the solution	TD	2000	[83]
66.	N-acetyl-tryptophan	N-acetyl-L-Trp N-acetyl-D-Trp	L: 17.1 ± 0.5 D: 12.7 ± 0.5	ΔK = 4.4 ± 0.7 α = 1.35 ± 0.07	ITC	298.15 K pH = 6.9	1:1	Added to the solution	TD	2000	[83]
67.	N-acetyl-tyrosine	N-acetyl-L-Tyr N-acetyl-D-Tyr	L: 130 ± 2 D: 125 ± 2	ΔK = 5 ± 2.83 α = 1.04 ± 0.02	ITC	298.15 K pH = 6.9	1:1	Added to the solution	TD	2000	[83]
68.	1-cyclohexyl ethylamine	(R)-1-cyclohexyl ethylamine (S)-1-cyclohexyl ethylamine	(R): 329 ± 3 (S): 328 ± 3	ΔK = 1.0 ± 4.2 α = 1.00 ± 0.01	ITC	298.15 K pH = 6.9	1:1	Added to the solution	TD	2000	[83]
69.	N,N-dimethyl-1- ferrocenylethylamine	(S)-N,N-dimethyl-1- ferrocenylethylamine (R)-N,N-dimethyl-1- ferrocenylethylamine	(S): 6700 ± 500 (R): 5600 ± 300	ΔK = 1100 ± 583 α = 1.20 ± 0.11	ITC	298.15 K pH = 4.8	1:1	Added to the solution	TD	2000	[83]
70.	2-phenylbutyric acid	(S)-2-phenylbutyric acid (R)-2-phenylbutyric acid	(S): 95 ± 2 (R): 94 ± 2	ΔK = 1.0 ± 2.8 α = 1.01 ± 0.03	ITC	298.15 K pH = 6.9	1:1	Added to the solution	TD	2000	[83]
71.	3-phenylbutyric acid	(S)-3-phenylbutyric acid (R)-3-phenylbutyric acid	(S): 430 ± 4 (R): 402 ± 4	ΔK = 28 ± 6 α = 1.07 ± 0.01	ITC	298.15 K pH = 6.9	1:1	Added to the solution	TD	2000	[83]
72.	propranolol	(S)-propranolol (R)-propranolol	(S): 117 ± 10 (R): 115 ± 10	ΔK = 2 ± 14 α = 1.02 ± 0.12	ITC	298.15 K pH = 4.8	1:1	Added to the solution	TD	2000	[83]
73.	Mandelic acid	(R)- mandelic acid (S)- mandelic acid	(R): 811 (S): 178	ΔK = 633 α = 4.56	NMR	303 ± 0.1 K pH = NP	1:1	Added to the solution	ROESY, molecular modeling	2000	[39]
74.	Hexahydromandelic acid	(R)-hexahydromandelic acid (S)- hexahydromandelic acid	(R): 3812 (S): 1040	ΔK = 2772 α = 3.65	NMR	303 ± 0.1 K pH = NP	1:1	Added to the solution	ROESY, molecular modeling	2000	[39]
75.	Brompheniramine (BrPh)	(+)-BrPh (-)-BrPh	(+): 822.7 (-): 798.5	ΔK = 24.2 α = 1.03	NMR	T = NP pH = 3.0	1:1	Added to the solution	ROESY, CE, UV–vis, ESI-MS, X-ray	2000	[40]
76.	Carvone (Crv)	(S)-(+)-carvone (R)-(-)-carvone	(S): 13.3 (R): 6.8	ΔK = 6.5 α = 1.96	NMR	T = 286 K pH = 7.0	1:1	Added to the solution	TD, molecular modeling	1999	[41]
77.	Verapamil (VP)	(+)-VP (-)-VP	(+): 272 ± 34 (-): 207 ± 59	ΔK = 65 ± 68 α = 1.30	NMR	NP	1:1	Added to the solution	CE, ESI-MS	1999	[42]
78.	Ketoconazole (KC)	(+)-KC (-)-KC	(+): 1146 ± 163 ^j^ (-): 750 ± 39 ^j^	ΔK = 396 ± 168 α = 1.53 ± 0.23	NMR	298 K pH = NP	1:1:1 ^j^	Added to the solution	API-MS, ROESY, molecular modeling	1999	[43]
79.	Ala-Phe	L-Ala-L-Phe D-Ala-D-Phe	LL: 14–40 DD: 12–42	ΔK = 2–5 α = 1.17–1	CE	298 K pH = 1.8–3.5	1:1	Added to the solution		1999	[60]
80.	Praziquantel (PZQ)	(+)-PZQ (-)-PZQ	(+): 335 (-): 224	ΔK = 111 α = 1.50	UV–vis phase solubility diagram	T = 310 ± 1 K pH = 7.0	1:1	Added to the solution	UV, DSC, X-ray, IR, API-MS	1998	[91]
81.	Bifonazole	S-bifonazole R-bifonazole	(S): 376–484 (R): 273–323	ΔK = 103–161 α = 1.38–1.50	RPLC	T = 293 and 278 K pH = 3.0	1:1	Added to the solution	TD	1998	[75]
82.	Econazole	S-econazole R-econazole	(S): 229–275 (R): 168–213	ΔK = 61–62 α = 1.36–1.29	RPLC	T = 293 and 278 K pH = 3.0	1:1	Added to the solution	TD	1998	[75]
83.	Sulconazole	S-sulconazole R-sulconazole	(S): 297–348 (R): 256–299	ΔK = 41–49 α = 1.16	RPLC	T = 293 and 278 K pH = 3.0	1:1	Added to the solution	TD	1998	[75]
84.	Miconazole	S-miconazole R-miconazole	(S): 57–68 (R): 38–45	ΔK = 19–23 α = 1.5	RPLC	T = 293 and 278 K pH = 3.0	1:1	Added to the solution	TD	1998	[75]
85.	Zileuton	(+)-zileuton (-)-zileuton	(+): 5752 ± 246 (NMR) (-): 5436 ± 735 (NMR) (+): 5866 ± 738 (UV) (-): 5686 ± 216 (UV) (+): 5071 ± 182 (CD) (-): 4508 ± 218 (CD)	ΔK = 316 ± 775 (NMR) α = 1.06 ± 0.15 (NMR) ΔK = 180 ± 769 (UV) α = 1.03 ± 0.14 (UV) ΔK = 563 ± 283.9 (CD) α = 1.13 ± 0.07 (CD)	NMR UV CD	T = 298 ± 1 K pH = NP	1:1	Added to the solution	ROESY	1998	[44]
86.	Propranolol	(S)-propranolol (R)-propranolol	S: 166 ± 13 R: 138 ± 26	ΔK = 28 ± 29 α = 1.20 ± 0.24	FS	T = NP pH = 2.5	1:1	Added to the solution	CE	1997	[73]
87.	Metomidate (MET)	(+)-MET (-)-MET	655/483	ΔK = 172 α = 1.36	NMR	T = NP pH = 3.5	1:1	Added to the solution	CE	1996	[29]
88.	Mianserin	(R)- mianserin (S)- mianserin	(2.59 ± 0.73)×10^2^/(1.35 ± 0.51)×10^2^	ΔK = (1.24 ± 0.89)×10^2^ α = 1.92 ± 0.90	CE	T = 298.15 K pH = 3.2	1:1	Added to the solution		1996	[57]
89.	Trimipramine	(R)- trimipramine (S)- trimipramine	(7.20 ± 2.40)×10^3^/(5.02 ± 1.33)×10^3^	ΔK = (2.18 ± 2.74) × 10^3^ α = 1.44 ± 0.61	CE	T = 298.15 K pH = 3.2	1:1	Added to the solution		1996	[57]
90.	Thioridazine	(R)- thioridazine (S)- thioridazine	(3.54 ± 3.02)×10^4^/(2.91 ± 1.43)×10^4^	ΔK = (6.30 ± 33.4)×10^3^ α = 1.22 ± 1.20	CE	T = 298.15 K pH = 3.2	1:1	Added to the solution		1996	[57]
91.	Thalidomide (THA)	(S)-(-)-thalidomide (R)-(+)-thalidomide	(S): 76 (R): 64	ΔK = 12 α = 1.19	HPLC	T = NP pH = 4	1:1	Added to the solution		1996	[81]
92.	Ephedrine	(1R,2S)-(-)-ephedrine (1S,2R)-(+)-ephedrine	(-): 79.2 ± 1.6 (+): 71.3 ± 1.2	ΔK = 7.9 ± 2.0 α = 1.11 ± 0.02	ITC	298.15 K pH = 5	1:1	Added to the solution	TD, NMR	1995	[85]
93.	Pseudoephedrine	(1S,2S)-(+)-pseudoephedrine (1R,2R)-(-)-pseudoephedrine	(+): 96.7 ± 1.0 (-): 68.9 ± 0.8	ΔK = 27.8 ± 1.28 α = 1.40 ± 0.02	ITC	298.15 K pH = 6.9	1:1	Added to the solution	TD, NMR	1995	[85]
94.	5-methoxy-3-(di-n-propylamino)chroman (5-MeO-DPAC)	(S)- 5-MeO-DPAC (R)- 5-MeO-DPAC	(S): 72 (R): 58	ΔK = 14 α = 1.24	CE	298.15 K pH = 7.0	1:1	Added to the solution		1995	[58]
95.	5-hydroxy-3-(di-n-propylamino)chroman (5-OH-DPAC)	(S)- 5-OH-DPAC (R)- 5-OH-DPAC	(S): 166 (R): 138	ΔK = 28 A = 1.20	CE	298.15 K pH = 7.0	1:1	Added to the solution		1995	[58]
96.	1-ferrocenylethanol (FET)	(+)-FET (-)-FET	(+): 1.26 × 10^3^ (-): 1.17 × 10^3^	ΔK = 0.09 × 10^3^ α = 1.08	Microcolumn LC	293 K pH = NP	1:1	Added to the solution		1994	[82]
97.	Dansylated-glutamate (Dns-Glu)	D-Dns-Glu L-Dns-Glu	D: 220 ± 4 L: 187 ± 4	ΔK = 32 ± 5.7 α = 1.18 ± 0.03	CE	298.15 K pH = 6.8	1:1	Added to the solution	HPLC	1994	[62]
98.	Dansylated-leucine (Dns-Leu)	D-Dns-Leu L-Dns-Leu	D: 170 ± 4 L: 141 ± 4	ΔK = 29 ± 5.7 α = 1.21 ± 0.04	CE	298.15 K pH = 6.8	1:1	Added to the solution	HPLC	1994	[62]
99.	Tioconazole	(+)-tioconazole (-)-tioconazole	(+) ^m^: (1.63–0.26) × 10^3^ (-) ^m^: (1.34–0.23) × 10^3^	ΔK ^m^ = 285–50 α ^m^ = 1.25–1.19	CE	298.15 K pH = 4.3	1:1	Added to the solution	NMR, TD	1994	[61]
100.	1,1′-binaphthol (BINOL)	(S)-1,1′-binaphthol (R)-1,1′-binaphthol	(S): 245 ± 4 (R): 176 ± 25	ΔK = 69 ± 25 α = 1.39 ± 0.2	FS	298.15 K pH = 5.5	1:1	Added to the solution	NMR, molecular modeling, CE, CD	1993	[74]
101.	1,1′-binaphthyl-2,2′-diyl hydrogen phosphate (BNP)	(S)-BNP (R)-BNP	(S): 542 ± 30 (R): 188 ± 20	ΔK = 354 ± 36 α = 2.88 ± 0.34	CD	298.15 K pH = 5.5	1:1	Added to the solution	FS, NMR, molecular modeling, CE	1993	[74]
102.	Dinitrophenyl (DNP)- amino acids: DNP-valine, DNP-leucine, DNP-methionine	D-isomer L-isomer	Details can be found in Section 7.7.7.	Details can be found in Section 7.7.7.	UV–vis CD NMR	273.15–298.15 K pH = 6.0 (UV–vis, CD) and pD = 11.0 (NMR)	1:1 (D-isomer); association of 1:1 complexes (L-isomer)	Added to the solution		1992	[45]

Abbreviations: API-MS—Atmospheric Pressure Ionization Mass Spectrometry; CD—Circular Dichroism; CE—Capillary Electrophoresis; DSC—Differential Scanning Calorimetry; ESI-MS—Electrospray Ionization Mass Spectrometry; FS—Fluorescence; FTIR-ATS—Attenuated Total Reflectance Fourier Transform Infrared Spectroscopy; FTIR—Fourier Transform Infrared Spectroscopy; GC—Gas Chromatography; GLC—Gas–Liquid Chromatography; HPLC—High-Performance Liquid Chromatography; HR-DOSY—High-Resolution Diffusion-Ordered Spectroscopy; HSCCC—High-Speed Counter-Current Chromatography; ITC—Isothermal Titration Calorimetry; LC—Liquid Chromatography; NA—Not Applicable; NOESY—Nuclear Overhauser Effect Spectroscopy; NP—Not Provided; ROESY—Rotating-Frame Overhauser Enhancement Spectroscopy; RPLC—Reversed-Phase Liquid Chromatography; RTP—Room Temperature Phosphorescence; TD—Thermodynamics; TG—Thermogravimetric Analysis; UV–vis—Ultraviolet Absorption Spectroscopy; * α was defined as the ratio of higher binding constant to lower one. ^a^ other approaches were also tested, including linear. ^b^ in the paper, binding constant was obtained for more than one temperature: 298, 303, 308 K. ^c^ chiral recognition not observed, binding constants have not been calculated. ^d^ binding constant determined for ternary complexes naproxen/β-CD/1-iodobutane. ^e^ binding constant determined for ternary complexes propranolol/β-CD/bromocyclohexane. ^f^ binding constant determined for ternary (for R-enantiomer) and quaternary complexes (for S-enantiomer) naproxen/β-CD/ 1,2-dibromoethane. ^g^ binding constant calculated from ΔG=-RTlnK, where R-gas constant, R=8.314 J/(K ∙ mol), T-temperature: T = 298 K. ^h^ the K values depend on the chosen H signal used to assess the binding constant, the experimental errors of determination of K values are less than 10%—^I^ unit not provided, the analysis provided in two steps: I—without β-CDs in the solvent, II—with addition of β-CDs to the mobile phase, the experimental errors of determination of K values are less than 5 and 10%, for native and poly-β-CD, respectively. ^j^ binding constant determined for ternary complexes ketoconazole/β-CD/L-tartaric acid. ^k^ K_H_ value calculated based on K_H_/K_L_ ratio and provided K_L_ value. ^l^_(+)_-amino acid in protonated form_, (±)_ -amino acid in zwitterionic form, _(-)_ -amino acid in anionic form. ^m^ the range of K, α, ΔK values depending on the concentration of methanol.

**Table 3:** The stability constants of complexes formed by the enantiomers with β-CD and IIRs [78].

M^−1^	CP		2,6-DKP		MPB	
β-CD	204.2	138.6	19.5	12.4	171.3	149.7
β-CD and C4	2.147 × 10^5^	1.493 × 10^5^	1.240 × 10^5^	9.688 × 10^5^	1.423 × 10^5^	1.256 × 10^5^
β-CD and C8	7.391 × 10^5^	5.657 × 10^5^	0.549 × 10^5^	0.336 × 10^5^	0.910 × 10^5^	0.761 × 10^5^
β-CD and C12	-	-	0.399 × 10^5^	0.251 × 10^5^	0.484 × 10^5^	0.413 × 10^5^

Cn indicates the length of the alkyl chain IIR, where n = number of carbon atoms.

**Table 4:** Conditions used to achieve the optimum enantioseparation for each drug [55]. Other conditions: the background electrolyte (BGE) pH was 2.5, phosphoric acid-triethanolamine buffer pH 2.5 + urea (if [β-CD] ≤ 20 mM, urea concentration = 2 M; if [β-CD] > 20 mM, urea concentration = 4 M; [β-CD]_opt_ (mM), optimum β-CD concentration; TEA- triethylamine, MeOH-methanol [55].

Chiral Drug	Buffer (mM)	T (°C)	[β-CD] (mM)	Modifier/Additive	K_1_ [M^−1^]	K_2_ [M^−1^]	ΔK [M^−1^] (α) α = K_H_/K_L_	[β-CD]_opt_ (mM)
Alprenolol	200	15	20	5% MeOH + 1% TEA	88.52	90.84	ΔK = 2.32 α = 1.03	11.15
Isoproterenol	100	20	50	1% TEA	7.31	11.89	ΔK = 4.58 α = 1.63	107.3
Isoxsuprine	100	30	15	0	57.73	58.72	ΔK = 0.99 α = 1.02	17.18
Metaproterenol	75	30	20	0	35.05	37.52	ΔK = 2.47 α = 1.07	27.58
Methoxamine	75	30	20	0	41.63	44.6	ΔK=2.97 α=1.07	23.21
Nadolol	100	15	50	0	11.38	11.65	ΔK = 0.27 α = 1.02	86.84
Pindolol	200	15	50	10%MeOH + 0.6% TEA	31.38	31.85	ΔK = 0.47 α = 1.01	31.63
Propranolol	75	15	20	20% MeOH	80.07	90.37	ΔK = 10.3 α = 1.13	11.76
Ritodrine	150	15	10	2% TEA	56.52	63.29	ΔK = 6.77 α = 1.12	16.72
Terbutaline	75	30	20	0	39.27	53.06	ΔK = 13.79 α = 1.35	21.91

**Table 5:** Apparent and averaged binding constants determined by CE (K in M^−1^) for the complexes formed between paliperidone, propranolol, risperidone or verapamil with the native β-CD or polyβ-CD at pH 2.5 and 7.0 [53].

	pH 2.5		pH 7.0	
	β-CD	Polyβ-CD	β-CD	Polyβ-CD
Paliperidone	167	437	193	574/602
Propranolol	157	247/273	144	517/537
Risperidone	91	562	216	729
Verapamil	308/350	1513/1643	410	1198/1332

Mean values calculated from three experiments. Relative standard deviations lower. than 5% with β-CD and than 10% with polyβ-CD.

**Table 6:** Stability constants [M^−1^] of the different β-CD complexes obtained from the potentiometric measurements depending on pH [93].

Amino Acid	A^-^·β-CD	A^-^·β-CD (HA^-^·β-CD) *	A ^±^ ·β-CD (H_2_A ^±^ ·β-CD)^*^	H_2_A^+^ ·β-CD (H_3_A^+^ ∙β-CD) *	H_3_A^2+^ ·β-CD
L-Phe + β-CD		116 ± 12	10 ± 3	6 ± 4	
D-Phe + β-CD		103 ± 14	10 ± 3	11 ± 5	
L-Tyr + β-CD	102 ± 10	84 ± 19	4.5 ± 2.5		
D-Tyr + β-CD	108 ± 12	120 ± 18	30 ± 9		
L-Trp + β-CD		86 ± 11	7 ± 3	2.5 ± 2.0	
D-Trp + β-CD		94 ± 13	19 ± 9	8 ± 7	
L-Leu + β-CD		21 ± 2	5 ± 4	3 ± 1.5	
D-Leu + β-CD		28 ± 3.5	~0	0.9 ± 0.4	
L-Asp + β-CD	3.4 ± 0.2	14 ± 1.5	11 ± 6	~0	
D-Asp + β-CD	11.3 ± 2.0	4.6 ± 0.3	1.5 ± 0.6	~0	
L-Glu + β-CD	5.7 ± 0.3	19 ± 2	13 ± 7	~0	
D-Glu + β-CD	2.7 ± 0.1	2 ± 1	1.1 ± 0.2	~0	
L-His + β-CD		3.3 ± 0.2	1.2 ± 0.5	0.6 ± 0.3	~0
D-His + β-CD		5 ± 1	5 ± 1	0.4 ± 0.1	~0
L-Thr + β-CD		2 ± 1	2 ± 1.5	2.5 ± 1.1	
D-Thr + β-CD		2.8 ± 0.6	0.9 ± 0.5	2.0 ± 1.2	

* For tyrosine, aspartic acid, and glutamic acid; A^-^ -deprotonated form of amino acid, A **^±^** -zwitterion.

**Table 7:** Dissociation constants (K_d_) for Inclusion Complexes of β-CD-DNP-Amino Acids [45] with calculated ratio of binding constant (α) for each pair of enantiomers.

Guest Molecules	K_d_ [M] (UV)	α (UV)	K_d_ [M] (CD)	α (CD)	K_d_ [M] (NMR)	α (NMR)
DNP-L.valine	(1.78 ± 0.10) × 10^−3^	1.33 ± 0.12	(2.83 ± 0.05) × 10^−3^	1.15 ± 0.03	2.85 × 10^−3^	1.19
DNP-D-valine	(2.36 ± 0.17) × 10^−3^		(3.26 ± 0.05) × 10^−3^		3.38 × 10^−3^	
DNP-L-leucine	(7.11 ± 0.41) × 10^−4^	1.62 ± 0.30	(1.66 ± 0.20) × 10^−3^	1.30 ± 0.22	1.52 × 10^−3^	1.6
DNP-D-leucine	(1.15 ± 0.21) × 10^−3^		(2.15 ± 0.25) × 10^−3^		2.43 × 10^−3^	
DNP-L-methionine	(1.38 ± 0.19) × 10^−3^	1.29 ± 0.22	(1.32 ± 0.07) × 10^−3^	1.64 ± 0.31	2.47 × 10^−3^	1.2
DNP-D-methionine	(1.78 ± 0.25) × 10^−3^		(2.16 ± 0.41) × 10^−3^		2.96 × 10^−3^	

UV measurements were made in 0.10 M sodium phosphate solution, pH 6.0; temperature, 25 °C; CD measurements were made in 0.10 M sodium phosphate solution, pH 6.0; temperature, 21 °C; NMR measurements were made in 0.10 M; sodium measurements were made in D_2_O containing 0.10 M sodium phosphate, pD 11.0; temperature, 20 °C [45].

## Abbreviations

**Abbreviation**	**Description**
2,6-DKP	2,6-diketopiperazine derivative
2′OHFL	(2-(2-hydroxy-phe-nyl)-2,3-dihydro-4h-chromen-4-one, 2′-hydroxyflavanone
4′OHFL	2-(4-hydroxyphenyl)-2,3-dihydro-4h-chromen-4-one, 4′-hydroxyflavanone
5-MeO-DPAC	5-methoxy-3-(di-n-propylamino)chroman
5-OH-DPAC	5-hydroxy-3-(di-n-propylamino)chroman
AA	2-acetyl-1-(4′-chlorophenyl)-6,7-dimethoxy-1,2,3,4-tetrahydroisoquinoline
ACE	Affinity Capillary Electrophoresis
Ala	Alanine
am-β-CD	6-amino-6-deoxy-β-cyclodextrin
ANP	Acyclic Nucleoside Phosphonate
ANP 4	(1-(1-(2,6-diamino-9h-purin-9-yl)-3-hydroxypropan-2-yl)-1,2,3-triazol-4-yl)phosphonic acid
ANP 5	2-((diisopropoxyphosphoryl)methoxy)-3-(2,4-dioxo-3,4-dihydropyrimidin-1(2h)-yl)propanoic acid
API	Active Pharmaceutical Ingredient
API-MS	Atmospheric Pressure Ionization Mass Spectrometry
Asp	Asparagine
ATR	Attenuated Total Reflection
BGE	Background electrolyte
BINOL	1,1′-binaphthol
BNP	1,1′-binaphthyl-2,2′-diyl hydrogen phosphate
BrPh	Brompheniramine
Cbz-	Benzyloxycarbonyl
CCDC	Cambridge Crystallographic Data Centre
CD	Circular Dichroism
CDs	Cyclodextrins
CE	Capillary Electrophoresis
CHMA	α-cyclohexylmandelic acid
CM-β-CD	Carboxymethyl-β-cyclodextrin
CP	Cyclopentolate
Crv	Carvone
CSP	Chiral Stationary Phase
CS	Chiral selector
CT	Citalopram
CZE	Capillary Zone Electrophoresis
de	Diastereomeric Excess
DIM	Dimethindene
DMF	N,N-Dimethylformamide
DNP-	Dinitrophenyl-
Dns-Glu	Dansylated-Glutamate
Dns-Leu	Dansylated-Leucine
DPAC	3,4-dihydro-2h-1-benzopyran
DSC	Differential Scanning Calorimetry
ee	Enantiomeric Excess
EOF	Electroosmotic Flow
ES	Enantioseparation
ESI-MS	Electrospray Ionization Mass Spectrometry
EurPh	European Pharmacopeia
FEP	Free Energy Perturbation
FET	1-ferrocenylethanol
FL	2-phenyl-2,3-dihydro-4h-chromen-4-onen, flavanone
FLUO	Fluorescence
FTIR	Fourier Transform Infrared Spectroscopy
FTIR-ATS	Attenuated Total Reflectance Fourier Transform Infrared Spectroscopy
FS	Fluorescence spectroscopy
GC	Gas Chromatography
GLC	Gas–Liquid Chromatography
Glu	Glutamic Acid
His	Histidine
HPLC	High-Performance Liquid Chromatography
HPMPA	9-[3-hydroxy-2-(phosphonomethoxy)propyl]adenine
HP-β-CD	(2-hydroxypropyl)-β-cyclodextrin
HR-DOSY	High-Resolution Diffusion-Ordered Spectroscopy
HSCCC	High-Speed Counter-Current Chromatography
IB	6-[imidazol-1-yl(phenyl)methyl]-3-methyl-1,3-benzothiazol-2-one
Ibu	1-iodobutane
ID	Inner Diameter
IIR	Ion Interaction Reagent
IR	Infrared
ITC	Isothermal Titration Calorimetry
K	Binding Constant
KC	Ketoconazole
KP	Ketoprofen
LC	Liquid Chromatography
Leu	Leucine
MeOH	Methanol
MET	Metomidate
MPB	Methylphenobarbital
NA	Not Applicable
N-Ac-	N-Acetyl-
Nap	Naproxen
NOESY	Nuclear Overhauser Effect Spectroscopy
NP	Not Provided
Phe	Phenylalanine
PMPA	9-[2-(phosphonomethoxy)propyl]adenine
PMPDAP	9-[2-(phosphonomethoxy)propyl]-2,6-diaminopurine
PTZ	Promethazine Hydrochloride
PZQ	Praziquantel
ROESY	Rotating-Frame Overhauser Enhancement Spectroscopy
RP-HPLC	Reverse-Phase High-Performance Liquid Chromatography
RPLC	Reversed-Phase Liquid Chromatography
RTP	Room Temperature Phosphorescence
SIB	Sibutramine
TAA	Tetraalkylammonium
TB	Tröger’S Base
TD	Thermodynamics
TEA	Triethylamine
TG	Thermogravimetric Analysis
THA	Thalidomide
Thr	Threonine
TM-β-CD	Heptakis(2,3,6-tri-o-methyl-)-β-cyclodextrin
Trp	Tryptophan
Tyr	Tyrosine
UV-Vis	Ultraviolet Absorption Spectroscopy
V_c_	Cavity volume
VP	Verapamil
Z-	Benzyloxycarbonyl
α ratio	Binding selectivity, ratio of binding constants
ΔK	Difference in binding constants
